# Revisiting the Taxonomic Synonyms and Populations of *Saccharomyces cerevisiae*—Phylogeny, Phenotypes, Ecology and Domestication

**DOI:** 10.3390/microorganisms8060903

**Published:** 2020-06-15

**Authors:** Ana Pontes, Mathias Hutzler, Patrícia H. Brito, José Paulo Sampaio

**Affiliations:** 1 UCIBIO, Departamento de Ciências da Vida, Faculdade de Ciências e Tecnologia, Universidade Nova de Lisboa, 2829-516 Caparica, Portugal; ap.pontes@fct.unl.pt (A.P.); phbrito@fct.unl.pt (P.H.B.); 2 Research Center Weihenstephan for Brewing and Food Quality, TU München, D-85354 Freising, Germany; m.hutzler@tum.de

**Keywords:** *Saccharomyces cerevisiae*, *Saccharomyces boulardii*, *Saccharomyces diastaticus*, population genomics, yeast domestication, *STA1*, *MEL1*

## Abstract

*Saccharomyces cerevisiae*—the most emblematic and industrially relevant yeast—has a long list of taxonomical synonyms. Formerly considered as distinct species, some of the synonyms represent variants with important industrial implications, like *Saccharomyces boulardii* or *Saccharomyces diastaticus*, but with an unclear status, especially among the fermentation industry, the biotechnology community and biologists not informed on taxonomic matters. Here, we use genomics to investigate a group of 45 reference strains (type strains) of former *Saccharomyces* species that are currently regarded as conspecific with *S. cerevisiae*. We show that these variants are distributed across the phylogenetic spectrum of domesticated lineages of *S. cerevisiae*, with emphasis on the most relevant technological groups, but absent in wild lineages. We analyzed the phylogeny of a representative and well-balanced dataset of *S. cerevisiae* genomes that deepened our current ecological and biogeographic assessment of wild populations and allowed the distinction, among wild populations, of those associated with low- or high-sugar natural environments. Some wild lineages from China were merged with wild lineages from other regions in Asia and in the New World, thus giving more resolution to the current model of expansion from Asia to the rest of the world. We reassessed several key domestication markers among the different domesticated populations. In some cases, we could trace their origin to wild reservoirs, while in other cases gene inactivation associated with domestication was also found in wild populations, thus suggesting that natural adaptation to sugar-rich environments predated domestication.

## 1. Introduction

*Saccharomyces cerevisiae*—the most emblematic and industrially relevant yeast species—was defined in 1838 by Meyen [1] and typified, i.e., linked to a living type strain, by Hansen in 1883. In the following decades, physiological and morphologic characters were the sole criteria available to yeast taxonomists and even minor phenotypic differences were considered adequate for species delimitations, a practice that promoted a continuous rise in the number of accepted species. As a consequence, Stelling-Dekker in 1931 [2], Lodder and Kreger-van Rij in 1952 [3] and van der Walt in 1970 [4] recognized 23, 30 and 41 *Saccharomyces* species, respectively. As in other microbial groups, yeast classification based on phenotypic markers was challenged when DNA based methods started to be implemented as taxonomic tools. As a result, numerous *Saccharomyces* species were recognized as synonyms of *S. cerevisiae* [5] and a list of approximately 100 species or varietal names was merged into that species. Such taxonomical changes were continued in subsequent decades and generated an understandable confusion among scientists and industry professionals not versed in yeast systematics.

More recently, complete genome sequencing has provided the necessary resolution to dissect *S. cerevisiae* at the population level and various studies have revealed a complex scenario with wild, domesticated and admixed groups [6,7,8,9]. Here, we use genomics to investigate a group of 45 reference strains (type strains) of former *Saccharomyces* species—currently regarded as synonyms of *S. cerevisiae*—many of which represent strains isolated from human-made fermentations worldwide. We show that these variants are distributed across the phylogenetic spectrum of domesticated lineages of *S. cerevisiae*, with a majority belonging to in the most relevant technological groups, but totally absent in wild lineages. We also examine salient features of domesticated lineages and reassess the biogeography and ecology of wild lineages.

## 2. Materials and Methods

### 2.1. Genome Sequencing, Read Alignment and Genotype Calling

Paired-end Illumina MiSeq (500 cycles) or NextSeq (300 cycles) reads were obtained for 37 strains. Genomic data for other strains were retrieved from public databases as indicated in Table S1. When only finished genome sequences were available, the corresponding error-free Illumina reads were simulated using DWGSIM.

Reads for each isolate were mapped to an extended *Saccharomyces* spp. reference containing sequences of *S. cerevisiae* (UCSC version sacCer3), *S. paradoxus*, *S. mikatae*, *S. kudriavzevii*, *S. uvarum* [10] and *S. arboricolus* [11], as previously described by [12] and using SMALT v. 0.7.5 aligner. The reference Index was built with a word length of 13 and a sampling step size of 2 (-k 13 -s 2). An intensive search for alignments (-x) was performed during the mapping step with the random assignment of ambiguous alignments switched off (-r -1) and the base quality threshold for the look-up of the hash index set to 10 (-q 10). With these settings, SMALT v. 0.7.5 only reports the best unique gapped alignment for each read. The insert size distribution was inferred with the ‘sample’ command of SMALT prior to mapping. Conversion of SAM format to BAM, sorting, indexing, several mapping statistics and consensus genotype calling were performed using the tools available in the SAMtools package v. 1.18 [13] and as described previously [14]. Multiple sequence alignments for each reference chromosome were generated from the resulting FASTA files. For downstream analysis, all bases with a Phred quality score below Q40 (equivalent to a 99.99% base call accuracy) or ambiguous base calls were converted to ‘N’.

### 2.2. Phylogenetic Analyses and Survey of Specific Genes

For the construction of the main phylogeny, if contributions from non–*S. cerevisiae* species were detected, only the *S. cerevisiae* subgenome was considered. Chromosomal single-nucleotide polymorphisms (SNPs) were extracted from a multiple sequence alignment only if the SNP was present unambiguously in at least 85% of the strains in the alignment. SNPs were then concatenated to generate a whole genome SNP alignment. Strains with more than 20,000 heterozygous sites with a Phred quality score above Q40 were selected for phasing. The BAM file of each strain with the paired-end read sequences mapped to the reference genome was analyzed with the phase command of SAMtools to infer both phases, thus solving the heterozygous SNPs. The -F option was used to exclude errors from unmapped or misaligned sequences. One haplotype per strain was randomly chosen and used in subsequent analyses. The main phylogeny was inferred using the maximum likelihood method as implemented in IQ-TREE v. 1.6.11 [15], using the TVM+F+ASC+G4 model of sequence evolution and the ultrafast bootstrap approximation with 1000 replicates [16]. The software iTOL v. 3.0 [17] was used for visualization. Single gene phylogenies were constructed with MEGA 7 [18], using the Tamura 3-parameter model and the maximum-likelihood method.

For the study of specific genes, whole genome assemblies were prepared with SPAdes v. 3.13.1. Prior to assembly, reads were processed with trimmomatic v. 0.36 to remove adapter sequences. In order to retrieve genes of interest, a local blast database was set for each genome and ORFS were searched with BLASTn using as queries: *AQY1* and *AQY2* of YPS163 [19], regions A, B and C of EC1118 [20], *RTM1*, *BIO1* and *BIO6* of CEN.PK13 [21], *MEL1* of UWOPS 03–461.4 and UWOPS 91–917.1 [22] and *STA1* [23]. Gene copy number was estimated by mapping the reads from each strain against the genome of strain UWOPS 03-461.4 [24], as described above. The median genome coverage was estimated as the coverage of each nuclear chromosome. Following a previous study [9], the ratio between the median coverage of each individual gene and the values of the genome coverage was considered a good estimate of the gene copy number.

### 2.3. Phenotypic Analyses

Strains investigated for the diastase phenotype were precultured in 20 mL of YPD (1% (*w/v*) yeast extract, 2% (*w*/*v*) peptone, 2% (*w*/*v*) dextrose) overnight at 25 °C. Washed cells were then inoculated in 50 mL of beer wort (11° Brix) and incubated for 15 days at 25 °C. The decrease of the °Brix was monitored and strains that in the end of the incubation period had a °Brix lower than 4 were considered positive for the diastase phenotype. Strains investigated for the ability to grow on melibiose were precultured in 200 µL of 0.2% (*w*/*v*) glucose and YNB (yeast nitrogen base, Difco) in a 96-well plate overnight at 25 °C. Then, 10 µL were inoculated 200 µL of 1% (*w*/*v*) melibiose and YNB. Growth was followed by measuring absorbance at 640 nm for three days in a Tecan Spark (Tecan Trading, Männedorf, Switzerland) microplate reader incubated at 25 °C.

## 3. Results

### 3.1. Global Phylogenetic Analysis Reveals an Unequal Distribution of Type Strains of Former Species

**Figure 1:**
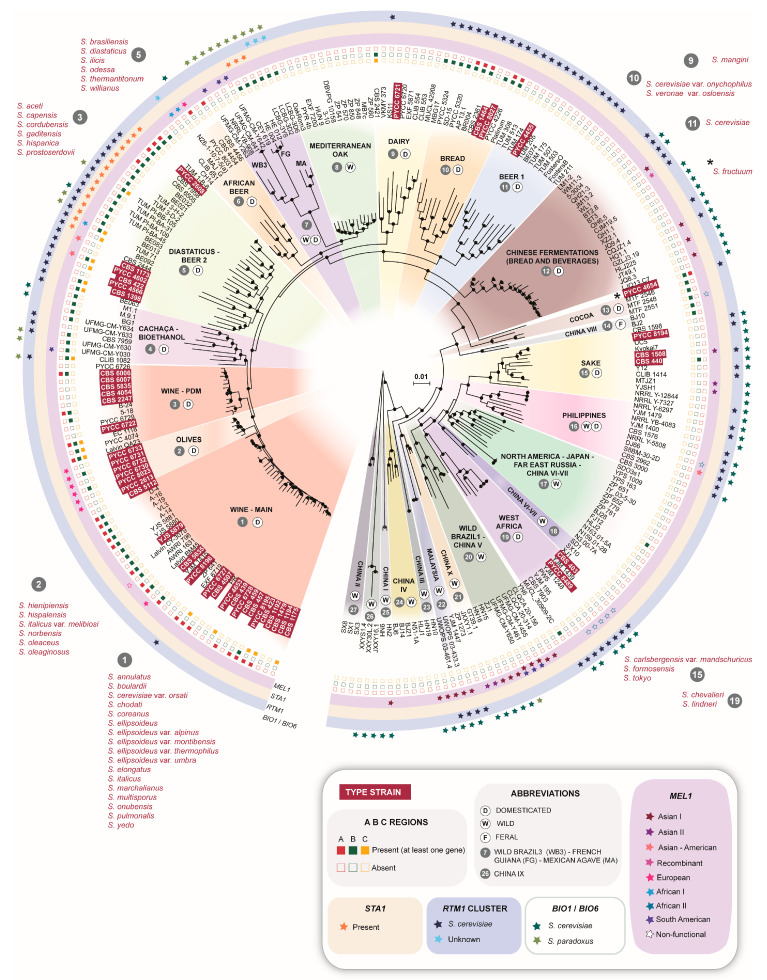
Current synonyms of *S. cerevisiae* are unequally distributed among the known lineages of the species. The phylogeny was inferred from 248 sequences and 1,520,302 single nucleotide polymorphisms using the TVM+F+ASC+G4 model of sequence evolution and the maximum likelihood method as implemented in IQ-TREE and was rooted with *S. paradoxus*. Branch lengths correspond to the expected number of substitutions per site and black dots in tree nodes depict bootstrap support values above 95% (1000 replicates). The 27 clades detected are numbered in gray circles, the former type strains of current synonyms of *S. cerevisiae* are highlighted in red rectangles.

We analyzed the genomes of 45 strains considered in the past as distinct *Saccharomyces* species (or varieties), but now regarded as *S. cerevisiae* synonyms, together with the valid type strain of *S. cerevisiae* (Table 1) and an additional 202 genomes representing the population diversity and technological variants currently known (Table S1). This is the most comprehensive dataset representing the diversity in this species. The phylogenetic analysis of these 247 genomes is shown in Figure 1 and a simplified unrooted phylogeny is shown in Figure S1. We identified 27 clades that represent the various technological variants of *S. cerevisiae* or geographical or ecological populations, many of which have been recognized previously by us or by other authors. In order to contribute to standardizing the designations of populations of *S. cerevisiae,* we provide a list of designations used here and a comparison with designations employed in other studies in Table 2. We also classified the 27 clades according to their association with domesticate or wild populations (Figure 1 and Table 2). In the first case the strains are associated with processes like wine or beer fermentations and are expected to have acquired specific domestication signatures. In the second case the populations have an ecological or geographic circumscription, like for example the Mediterranean oaks population.

Except for PYCC 4654, the type strain of *S. fructuum*, that occupies an isolated position in the phylogeny outside any of the recognized clades, all other type strains formed clades with technological, and therefore domesticated groups, namely the Wine (two clades), Olives, Beer (two clades), Bread, Dairy, Sake and West African groups (Figure 1). Therefore, the distribution of type strains of former species is markedly unbalanced across the spectrum of genetic diversity of *S. cerevisiae.* The Wine and Olives groups congregated most (29 out of 45) of the type strains analyzed, followed by the two beer groups that together gathered seven type strains, including the recognized type strain of *S. cerevisiae* (PYCC 4455), a beer strain assigned to the Beer 1 clade. Overall, nine of the 27 clades that represent distinct populations of *S. cerevisiae*, included at least one type strain. Remarkably, all the wild populations like those found in arboreal niches in China, Europe or the Americas did not include any of the type strains of former species. A more detailed analysis of the distribution of former *Saccharomyces* type strains in the context of the groups to which they were assigned is presented below.

### 3.2. The Wine and Olives Clades Harbor Most Type Strains of Former Species

The largest number of type strains of former *Saccharomyces* species (23) was assigned to the two clades that congregate wine yeasts (clades 1 and 3, the main Wine clade and the Prise de Mousse clade, respectively). Most of these former type strains (16) were isolated from wine or grapes and two strains are human clinical isolates. The remaining strains were isolated from other fruits (2) or other fermented beverages (2) and one strain has an unknown origin. Previous studies have revealed that an important feature of the Wine clade is the inclusion of strains exhibiting typical genomic markers of a wine strain, but that were isolated in a clinical setting, mostly as human opportunists [7,9]. The two former type strains mentioned above were isolated from similar settings, *S. annulatus* from a human skin infection in 1929 and *S. pulmonalis* from the sputum of a tuberculosis patient in 1925, thus confirming that the association between clinical and wine strains is, at least, one century old. Another clinically relevant strain is the reference strain of S. *boulardii,* widely employed as a probiotic [35]. This synonym of *S. cerevisiae* was isolated from a lychee in Vietnam but has the main genomic features of a wine strain (Figure 1, Table S1). Therefore, this clade includes both clinical and probiotic strains, which do not have clear discernible features at the genome level.

As already mentioned, all the former type trains isolated from wine investigated here joined clades 1 or 3. Clade 3 gathers a particular type of wine yeasts relevant for Jerez (Sherry) wines, a type of wine that undergoes an aging process in which an adapted variant of wine yeasts, the flor yeasts, forms a surface biofilm and contributes to the production of specific flavors and aromas [36]. Other genomically similar strains isolated in other countries like Hungary, France or Italy, are particularly resistant to the stresses associated with the advanced stages of fermentation. Probably due to this, commercial strains from this group like the well-known EC1118 [20], proved to be adequate for secondary fermentations in champagne, a process also designated as “prise de mousse” [37] or to re-start stuck fermentations. Four of the six former type strains associated with the prise de mousse clade were isolated in Spain and the remaining two in Armenia and South Africa. The Prise de Mousse clade includes also Georgian wine strains (Figure 1, Table S1) isolated from amphorae [9]. It was recently hypothesized that since the Caucasus is thought to be the birthplace of winemaking, these Georgian isolates could represent the closest relatives of the first domesticated wine strains [9]. However, not only other Georgian strains cluster in the main wine clade and not in the Prise de Mousse clade (Figure 1, Table S1), but, as indicated above, several other strains of different origins and with highly specialized phenotypes are part of the Prise de Mousse clade, that therefore may be derived instead of ancestral.

As previously discussed [20,25,29,38], three genomic regions usually designated as A, B and C, were horizontally transferred from non-*Saccharomyces* yeasts. They encompass 39 genes potentially relevant for the winemaking process. These regions are well-represented in clades 1 and 3 (Figure 1). In line with this, these regions were also more prevalent in the type strains that joined the two wine clades than in type strains that joined other clades.

Six former type strains, mostly isolated from alpechin, the wastewater of olive oil mills, were assigned to a clade distinct, but related to those of wine yeasts (Figure 1, Table 1, Table S1). These strains are *S. cerevisiae* x *S. paradoxus* hybrids [26] and their ecological niche is related to processed olives, including table olives, olive oil and waste water from olive oil mills. The *S. cerevisiae* subgenomes of these strains (the *S. paradoxus* subgenome was excluded from the analysis) cluster close to the main wine clade (Figure 1), which indicates that the *S. cerevisiae* ancestor of these hybrids was a wine strain. The species to which these strains were associated with were described between 1957 and 1978 (Table 1) and all had the peculiarity of being unable to assimilate sucrose, an uncommon feature for *S. cerevisiae*. We confirmed that in all strains of the Olives clade, *SUC2* has a premature stop codon, thus explaining the observed phenotype. Another peculiar trait was the capacity to assimilate melibiose, again an uncommon trait for *S. cerevisiae.* Traditionally, melibiose utilization was regarded as a distinctive marker for the discrimination of Mel^−^ *S. cerevisiae* wine and top-brewing yeasts from Mel^+^ *S. pastorianus*, the bottom-brewing yeasts.

### 3.3. The Beer and Bread Clades Contain Multiple Type Strains of Former Species

**Figure 2:**
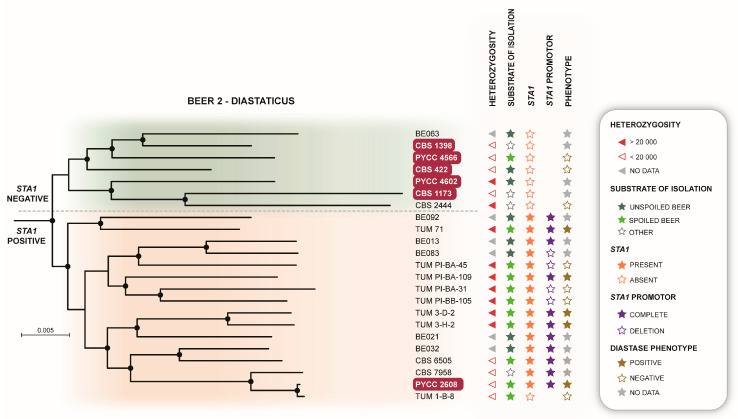
The Beer 2-Diastaticus clade of *S. cerevisiae* contains *STA1*-positive and *STA1*-negative strains. Detail of the main phylogeny showing the phylogenetic relationships within the Beer 2-Diastaticus clade together with relevant genetic and phenotypic attributes.

*S. cerevisiae* ale beer yeasts are assigned to two main clades, Clade 11 (Beer 1) that includes most beer types (British, German, wheat beer and Belgian beers) and Clade 5 (Beer 2) that includes low-gravity Saison-type beers (Figure 1). Whereas the main beer group (Beer 1) included only one type strain, in fact the valid type strain of *S. cerevisiae*, which was isolated from a brewery in the Netherlands, the Beer 2 clade included six type strains of former species. Not surprisingly, most type strains in this clade were isolated from beer and the most notable one is the type strain of *S. diastaticus*. This species was described more than 60 years ago as an unusual *S. cerevisiae*-like yeast occurring preferentially as a contaminant in beer fermentations [39,40]. The diastase (starch-degrading) ability of *S. diastaticus* is encoded by the chimeric gene *STA1* that gives rise to an extra-cellular glucoamylase that allows the conversion of soluble starch and dextrin into fermentable sugars. This leads to an abnormal and undesirable attenuation of beer, corresponding to a specific gravity much lower than what is typical for most beers [41]. Our assignment of the type strain of *S. diastaticus* to the Beer 2 clade is in line with a recent report indicating the association of diastase-positive strains with this clade [23]. Adding to the elucidation of the contribution of *STA1* and its polymorphisms to desirable and undesirable brewing properties and its association mostly with the Beer 2 clade [23], we provide here the phylogenetic placement of seven additional beer-spoiling strains. The phenotypes of these strains were recently characterized [42], and here they were all assigned to the Beer 2 clade (Figure 1). Therefore, this clade, that we rename as “Beer 2-Diastaticus” (Table 2), combines not only strains adequate for beer production, but also an important group of beer-spoilage yeasts. Most notably, these two technologically distinct yeast types are not easy to differentiate at the genome level since brewing strains and spoilage strains appear intermingled in a detailed view of the Beer 2-Diastaticus clade (Figure 2). Interestingly, several of the members of this clade had an excess of heterozygous sites which are likely an indication of ploidy levels higher than 2n (Figure 2), as already shown for Beer 1 strains that are tetraploid [30,43]. However, contrary to Beer 1 and bread yeasts, which invariably have a ploidy higher than 2nd, the Beer 2-Diastaticus clade appears to contain both 2n and >2n strains, which is an uncommon situation among the different populations of *S. cerevisiae* known so far. Our results are in line with previous findings on the strongly variable nature of the diastase activity [23]. Moreover, they support the division of the Beer 2-Diastaticus clade into two subclades, one where the presence of *STA1* could not be detected (subclade *STA1*-negative) and another one positive for *STA1*. The *STA1*-negative subclade congregates most of the former type strains found in this group, some of which are associated with brewing or with beer deterioration, but not with diastase activity (Figure 2). The *STA1*-positive subclade harbors the type strain of *S. diastaticus* and all diastase-positive spoilage strains, together with several brewing strains. A 1162-bp deletion in the promoter of *STA1* has been shown to explain a weak diastase activity in *STA1*-positive strains [23]. This could explain the occurrence on the same lineage of spoiling and beer-production strains. Our survey of this deletion among the genomes of the *STA1*-positive subclade revealed that all the five strains that exhibited a diastase activity in vitro (Figure S2) had the promoter region intact, whereas the diastase-negative strains for which *STA1* was detected (three strains) had the deletion (Figure 2).

The Bread clade contains strains used for bread leavening and is closely related to the beer yeasts of the Beer 1 clade. It includes two former type strains that are clinical isolates, *S. cerevisiae* var. *onychophilus* and *S. veronae* var. *osloensis*. This suggests that besides the Wine clade, the Bread clade also hosts opportunistic strains.

### 3.4. Additional Clades Harboring Type Strains of Former Species

The remaining six former type strains are placed in three additional clusters, all associated with human-made fermentations (Figure 1). The type strain of *S. mangini*, found in cheese, was associated with the Dairy clade (Clade 9). This clade was recently described [25] and gathers strains exclusively isolated from dairy products. Three other former type strains belonged to the Sake clade (Clade 15) that gathers strains used to ferment sake in Japan and other cereal-based beverages in other parts of Asia such as Laos, Philippines and Tibet. Not surprisingly, the former type strains of the Sake clade were isolated in China, Japan and Taiwan from sake and other fermentations. Finally, two reference strains clustered in the West Africa clade (Clade 19), and both were isolated from African artisanal fermented beverages, one from ginger beer (*S. lindneri*) and the other from palm wine (*S. chevalieri*). For this last species we sequenced strain PYCC 8489, obtained from the NRRL collection (NRRL Y-12633) in 2009. We found its genome sequence to be markedly distinct from that of a supposed copy of NRRL Y-12633, strain Y12 [44,45]. Whereas PYCC 8489 clustered in the West African clade, Y12 belonged to the Sake clade (Figure 1). Given the isolation of *S. chevalieri* from an African fermented beverage, we believe that the sequence of PYCC 8489 represents the correct placement of *S. chevalieri,* whereas Y12 probably does not represent the original African isolate.

### 3.5. The Biogeography and Ecology of Wild Populations

Although the recognition and study of wild populations of *S. cerevisiae* is of major importance for the detailed understanding of the emergence of domesticates, our knowledge of wild lineages and of their natural biology is much less advanced than that of domesticated populations and their features. In our analysis, we identified 13 wild clades that therefore represent almost half of the total of 27 clades recognized (Figure 1). Our main criteria for identifying a wild clade are the ecological association with a natural niche, sometimes coupled with a well-defined geographical distribution, and the absence of domestication signatures (see below). In two cases, we characterized a clade simultaneously as wild and domesticated. Clade 7 combines wild strains from Brazil, aboriginal human-associated strains from French Guiana and Mexican agave fermentation strains. Clade 16 combines strains directly isolated from fruits or tree sap and palm wine strains. In one situation (Clade 14) we characterized a clade as feral, since while its members originate in an arboreal wild environment in China, they possess several typical domestication signatures (Table 2). The wild clades did not include any of the former type strains, thus emphasizing their recent discovery.

Some wild lineages considered here result from the combination of previously recognized lineages. The most striking example is Clade 17, which combines the North America-Japan group [8], the Far East Russia group [9] and the China VI–VII groups [33]. In this case, a wild population predominantly associated with the arboreal niche spans from China to Far East Russia, Japan and North America. Other cases involve the already mentioned Clade 7 in Central and South America and Clade 20 in China, Ecuador and Brazil.

**Figure 3:**
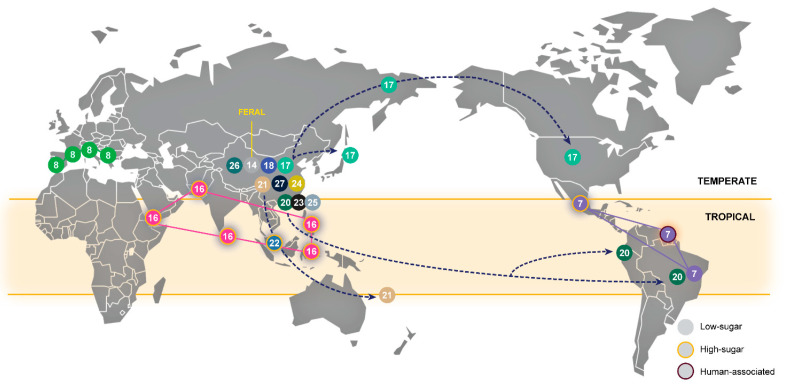
The global biogeography and ecology of wild populations of *S. cerevisiae*. Populations are numbered and color-coded according to the clades depicted in Figure 1. Circles without outline color correspond to populations associated with low-sugar environments and circles outlined in orange correspond to populations associated with high-sugar environments (in the case of Clade 7, the violet outline color indicates association to the human gut). Possible migration routes are indicated with arrowed dashed lines and population ranges are depicted with solid lines.

Figure 3 summarizes our current understanding of the biogeography and ecology of wild populations of *S. cerevisiae*. As already discussed [34], most wild lineages are Chinese or have been originally detected in China. Seven of the 13 wild lineages are exclusively composed of Chinese strains and three additional lineages contain strains from China and from other regions (Figure 3). Moreover, most wild lineages (10 lineages) are associated with arboreal niches, like tree bark, decaying wood and soil underneath trees. In such habitats simple sugars are scarce and therefore the “make-accumulate-consume” (ethanol) ecological strategy of *S. cerevisiae* [46], may not take place. On the contrary, the Malaysian population (Clade 22), for which a very limited number of clonally related isolates is known, appears to fulfill the anticipated ecological profile of a wild *S. cerevisiae* population, since it thrives in a sugar-rich environment where ethanol accumulates [47]. Additional wild populations from sugar-rich environments are scarce. The Philippines clade (Clade 16) is a candidate wild population since it contains strains isolated from sap or fruits of palm trees (Table S1). However, several strains in this clade came from different kinds of palm wine, which could suggest a domesticated origin.

The populations found in low-sugar niches appear to occur predominantly in temperate climates and the populations found in high-sugar niches seem to be associated with tropical regions (Figure 3). In China, three wild populations from low-sugar environments were found in the limit of the tropical zone and one of them (Clade 20) is also found in tropical South America (Brazil and Ecuador) (Figure 3). The other wild neotropical population (Clade 7) has a complex ecology and is associated with low- and high sugar environments, and also with the human gut (Figure 3).

### 3.6. Lifestyle Drives the Fate of Aquaporins

The loss of function of aquaporins, which are membrane water channels that facilitate the transport of water in and out of the cell, is viewed as a consequence of domestication. Functional aquaporins, encoded by the genes *AQY1* and *AQY2*, decrease fitness in environments with a high osmolarity caused by an elevated concentration of sugars, like wine must or beer wort, although they contribute to the fitness of wild strains [19]. We observed that all the wild populations associated with the low-sugar arboreal niche (Figure 3) have functional aquaporins (Table S1), while in the Wine, Beer 1, Sake and other domesticated clades the inactivation of aquaporins is the norm [29,43] (Table S1). As discussed elsewhere, the inactivating mutations of *AQY1* and *AQY2* are distinct across the various populations [43], which suggests that the loss of function occurred independently and multiple times. The Wine and related secondarily domesticated populations like the Cachaça-Bioethanol population, and also the Dairy population, share the same type of aquaporin gene inactivation, while the Sake and Chinese Fermentations populations exhibit other types of inactivating mutations (Table S1). Moreover, we also observed the inactivation of aquaporins in wild populations associated high-sugar environments, namely the Malaysian and Philippines populations (Table S1). Interestingly in Clade 7, found in the neotropics, the subclade associated with the arboreal niche in Brazil has functional aquaporins whereas the subclades associated with the human gut (French Guiana) and agave fermentations (Mexico) have non-functional aquaporins, whose genes exhibit unique mutations within each subclade (Table S1). Whereas the Malaysian and neotropical populations have distinctive inactivating mutations, not found in any other population, the inactivating mutations in the Philippines population (Clade 16) are the same as those found in the Sake (Clade 15) and Chinese Fermentations (Clade 12), which could indicate a wild—domesticate relationship in the Asian region. Taking into consideration this broader population-level analysis, the argument that aquaporin genes are maintained in two states by balancing selection [19] gets additional support. Moreover, it may be hypothesized that in *S. cerevisiae* aquaporin gene inactivation occurs whenever a transition to a high-sugar niche occurs, thus including the occurrence of inactivation in wild populations. Therefore, this loss of function may predate human domestication and may be a general and natural response of *S. cerevisiae* to nutritionally rich environments that also occurs, independently and with distinct mutations, in different domesticated lineages.

### 3.7. The Natural Reservoirs of RTM1 and BIO1/BIO6

*RTM1* is a subtelomeric gene associated with the locus of sucrose utilization and provides resistance to inhibitory compounds present in molasses [48]. This gene tends to be present in domesticated populations that are grown in molasses or equivalent substrates like beer wort, sugar cane juice or dough, but not in wine yeasts [49]. *RTM1* can be viewed as a domestication signature of several clades (Figure 1), and twelve type strains contained this gene, including the valid type strain of *S. cerevisiae* (Beer 1 clade), five former type strains in the Beer 2-Diastaticus clade, and another six type strains distributed in the Bread, Sake and West Africa clades. The population analysis of *RTM1* occurrence shows that it is also present in wild strains, like those of the Philippines (Clade 16), China X (Clade 21) and Malaysia (Clade 22), thus suggesting that domesticated strains may have acquired this gene from natural reservoirs in wild populations. A distinct *RTM1* allele was found in strains of the African beer population (Clade 6) and in strains used to ferment Mexican agave (Clade 7). Given the absence of *RTM1* homologs in *S. paradoxus*, it is difficult to access at this stage if these divergent sequences are intraspecific *S. cerevisiae* alleles or if they have a foreign origin.

*BIO1* and *BIO6* encode enzymes involved in the synthesis of biotin and were considered to have a restricted distribution in *S. cerevisiae*, being present in strains used in sake fermentation, but absent in wine strains [43,49,50]. More recently, *BIO1*/*BIO6* were found to be present in cachaça strains and in wild Brazilian strains [29]. Here, we detected *BIO1*/*BIO6* in 15 of the 27 clades depicted in Figure 1. North American *S. paradoxus* alleles of *BIO1*/*BIO6* appear to have introgressed into American populations. Earlier we reported such alleles in wild Brazilian populations (Clade 7 and Clade 20) and in the Cachaça-Bioethanol group (Clade 4) and also in supposedly European brewing strains of the Beer 2-Diastaticus clade [28]. Here we confirmed and expanded those observations and detected the same type of *S. paradoxus* alleles in additional representatives of the Beer 2-Diastaticus clade and also in all representatives of Clade 7. As discussed above for *RTM1*, wild populations in Asia (China, Malaysia, Philippines) may have been the original reservoirs of *BIO1/BIO6* that later disseminated into domesticated lineages propagated in substrates where biotin is scarce, like sake must.

### 3.8. The Complex Distribution of MEL Alleles

The utilization of melibiose requires the expression of an α-galactosidase that hydrolyzes this disaccharide into glucose and galactose. This property is encoded by a set of polymorphic *MEL* genes [51,52,53] that are present in *S. eubayanus*, *S. uvarum* and *S. mikatae,* but that are rare in *S. cerevisiae* and *S. paradoxus* [54,55]. In a series of studies, Naumov and coworkers characterized by genetic mapping 15 genes (*MEL1* to *MEL15*) in *S. cerevisiae* [55,56], that were found to be distributed in 11 chromosomes in telomeric regions. Together, these studies revealed that wine strains rarely contained this gene, but that strains related to the niche of processed olives could have multiple, highly similar, *MEL* genes. Another study reported that the Malaysian population (Clade 22) had a good ability to utilize melibiose [22]. This study also revealed a case of melibiose utilization associated with the West African population (Clade 19), but with a distinct evolutionary history since the West African *MEL* gene appeared to represent an introgression from *S. paradoxus*.

**Figure 4:**
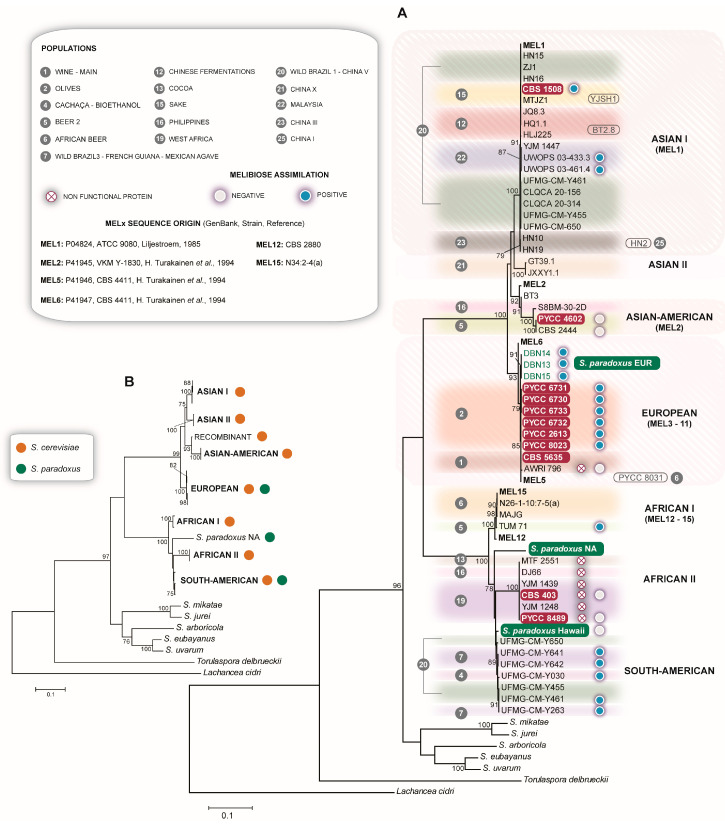
Phylogenetic analysis of *MEL* alleles of *S. cerevisiae.* (**A**) Phylogenetic overview of *MEL* alleles from 16 populations of *S. cerevisiae*. For comparison, *MEL* sequences from other *Saccharomyces* species were included together with *S. cerevisiae MEL* alleles (*MEL1*, *MEL2*, *MEL5*, *MEL6*, *MEL12* and *MEL15*) retrieved from the literature and from the NCBI database (*MEL12* and *MEL15* were retrieved directly from genomic assemblies). For *S. paradoxus*, sequences from representatives of the European (EUR), North American (NA) and Hawaiian populations were added. Incomplete sequences that could be assigned to an allelic group, but that were not used in the final phylogeny are indicated next to the tree in a rounded rectangle. The former type strains of *S. cerevisiae* are indicated in red rectangles; (**B**) simplified phylogeny of *MEL* alleles in the genus *Saccharomyces*. The two phylogenetic trees were constructed using the Maximum Likelihood method and the Tamura 3-parameter model. Bootstrap values > 75% are indicated (1000 replicates).

Our analysis indicated that, although infrequent within the phylogenetic spectrum of *S. cerevisiae*, *MEL* occurrence is wider, and its divergence is more complex, than previously anticipated. Of the 27 clades of *S. cerevisiae* depicted in Figure 1, 16 contained *MEL* alleles. However, the frequency of MEL^+^ genomes in those 16 clades was in most cases lower than 50% and only in two cases (considering only clades with five or more sequences), the complete fixation of the *MEL* gene was observed (Olives and Wild Brazil 1-China V). Moreover, five of the 16 clades with MEL genes included former type strains. From the phylogenetic comparison of all *MEL* sequences detected, we inferred that the native *S. cerevisiae* sequences could be resolved into three alleles, with four additional alleles of uncertain origin (Figure 4A). One allele, “Asian I” (*MEL1*), was present in genomes assigned to six clades that have in common an Asian origin. This allele was present in several strains of the Chinese Fermentations clade (Clade 12) and of the Sake clade (Clade 15), including the type strain of *S. carlsbergensis* var. *mandschuricus* that belongs to this last clade (Figure 4A). This allele was also present in wild populations: Wild Brazil 1-China V (Clade 20), Malaysia (Clade 22), China III (Clade 23) and China I (Clade 25). Another allele, “Asian II”, was present in China X (Clade 21). A third allele, “Asian-American”, was found in an Asian strain isolated in Sri Lanka and belonging to the Philippines clade (Clade 16) and in two genomes of the Beer 2-Diastaticus clade (Clade 5). These two Asian-American alleles were found in the type strain of *S. brasiliensis*, a Brazilian brewing strain and in a North American distillery strain. Recombination between Asian II and Asian-American alleles was detected in strain BT3, a Chinese domesticated strain from Clade 12. A similar recombinant allele corresponds to *MEL2* found in a strain isolated from dewberries in Russia [56]. A fourth *MEL* allele, “European”, was detected in two wine strains of Clade 1 (Wine) and in all strains of Clade 2 (Olives). In line with the information available in the literature, we confirmed that in wine strains (clades 1 and 3) the occurrence of this gene is rare, as we did not detect additional *MEL* genes in wine strains. The two genomes that had the *MEL* genes were that of CBS 5635, the type strain of *S. coreanus* and the genome of the commercial wine strain AWRI 796, but in this case the gene had inactivating mutations. Curiously, both strains were isolated in South Africa and another South African strain, from millet beer (Clade 6), also had this allele. A survey in our database of 170 genomes of *S. cerevisiae* wine strains revealed that 10 additional genomes contained *MEL* genes, but only in two cases the genes were functional. In contrast, all the strains of the Olives clade, i.e., six former type strains, were MEL^+^. Even in a larger strain dataset of more than 20 genomes of this clade related to a previous study [26], we could detect the presence of *MEL* in all strains, which makes the contrast with *MEL* scarcity in the wine clades even more striking. This allele corresponded to several *MEL* genes described previously, from *MEL3* to *MEL11* [56], which appear to represent a recent event of gene expansion in *S. cerevisiae* associated with domestication. All strains of the Olives clade had four or more *MEL* copies, with the highest copy numbers being recorded for *S. hienipiensis* (10) and *S. norbensis* (11). Interestingly, the European allele was also found in three strains of *S. paradoxus* (Figure 4A). These strains were isolated in Greece and belong to the European population of that species. The *S. cerevisiae* and *S. paradoxus* sequences of the European allele are almost identical, which suggests a case of introgression between these two species, but whose donor and recipient cannot be unequivocally identified at present.

A fifth allele (African I), is found mostly in *S. cerevisiae* strains isolated in Africa (Clade 6, African beer) and corresponds to *MEL12* to *MEL15* [51]. A similar allele (African II) is also present in African strains, mostly of Clade 19 (West Africa), but is a pseudogene, as previously reported [22]. The type strains of *S. chevalieri* and *S. lindneri* contain this allele. The last allele (South American) is present in wild Brazilian strains (clades 7 and 20) and in Brazilian domesticated strains used for sugar cane (cachaça) fermentations (Clade 4). Interestingly, Clade 20 that combines wild Chinese and wild Brazilian and Ecuadorean strains, contains the Asian I allele, present in all strains and the South American allele that co-occurs with the Asian I allele in Brazilian strains.

The African and South American alleles are not only considerably divergent from the Asian and European ones, but also share a phylogenetic proximity with *S. paradoxus* MEL genes found in representatives of the North American and Hawaiian population of this species (Figure 4A). Again, an introgression involving *S. cerevisiae* and *S. paradoxus* can be hypothesized, but the donor and the recipient species cannot be identified at present. The frequency of MEL genes in *S. paradoxus* appears to be low. Among the European strains, we surveyed 40 genomes and only in three cases did we found these genes. For the other populations we detected two positive cases among 30 surveyed genomes (Table S2).

We tested strains harboring distinct alleles for the ability to grow on melibiose and observed some cases of absence of growth on this compound (Figure 4A and Figure S3). As expected, the inactivating mutations in the European allele of wine strains and in the African II allele resulted in a MEL^-^ phenotype. Moreover, the strains with the Asian-American alleles and the North American and Hawaiian strains of *S. paradoxus* were also incapable of growing on melibiose in spite of having apparently functional genes.

A simplified phylogeny of the *MEL* gene for the genus *Saccharomyces* is presented in Figure 4B. Given the suspected introgressions to or from *S. paradoxus*, the boundaries between *S. cerevisiae* and *S. paradoxus* are not easily discernible. Since the average genome divergence between *S. cerevisiae* and *S. paradoxus* is 10%, the approximate 10% pairwise sequence divergence observed between the Asian I, II and Asian-American and the European allele could represent the above mentioned species-level divergence (Figure 4B). In this scenario, Asian I, II and Asian-American would represent the *S. cerevisiae* native allele and European would represent the *S. paradoxus* allele. Consequently, the European alleles found in *S. cerevisiae* wine and olives strains would represent an introgression from *S. paradoxus*. However, the origin of the remaining African and South American alleles, which are 20% divergent from the Asian I allele, is puzzling and can only be explained by an introgression from an unknown *Saccharomyces* species that is phylogenetically more related to *S. cerevisiae* and *S. paradoxus* than any currently known *Saccharomyces* species. An alternative explanation is to view the Asian I, II, Asian-American and the European alleles as native to *S. cerevisiae*, in spite of their remarkable divergence, and the African-South American ones as native to *S. paradoxus*. In this scenario, *MEL* divergence between *S. cerevisiae* and *S. paradoxus* is approximately double the average genomic divergence. This hypothesis also implies that the European allele was introgressed from *S. cerevisiae* into *S. paradoxus* and the African-South American alleles were introgressed from *S. paradoxus* into *S. cerevisiae.*

## 4. Discussion

In this study, we attempted to compare the diversity of a group of 45 type strains of species now viewed as *S. cerevisiae* synonyms, while simultaneously reassessing the population landscape of the species at a global scale. Under the lens of genomics, this group of former type strains represents a valuable historical record of diversity, ecology and biogeography that spans from 1870 (*S. ellipsoideus*) to 1984 (*S. boulardii*). When this diversity is superimposed on the global population diversity of *S. cerevisiae*, former type strains map reasonably well on the known domesticated populations but are absent in wild populations. This is an eloquent illustration of the roots of our biased understanding of *S. cerevisiae* primarily as a utility in wine, beer and bread production (among much other fermented products), rather than as a microbe shaped by its natural history. Another interesting observation is the strong redundancy of old species delimitations, based mostly on strains that belong to the Wine and Olives populations. A minor redundancy is also present in the Beer 2-Diastaticus, Bread, West Africa and Sake clades, but not in the Dairy and Beer 1 population. Our analysis also clarifies the status of historically and technologically relevant *Saccharomyces* species names for users in the fermentation industry.

Various population genomics studies of *S. cerevisiae* have been published in recent years and consequently distinct designations have been used to name the same population. Such lack of consistency, together with incomplete sampling, strong redundancy of certain domesticated lineages or the improper use of the “wild” descriptor, call for the need of an informed debate. Population VIII from China (Clade 14) provides a striking example of the difficulties in recognizing a wild lineage. Undisputedly considered as “wild”, it was even used as evidence supporting the East Asian origin of all domesticated *S. cerevisiae* strains since it carries duplicated genes involved in maltose metabolism, thus appearing as the direct link between wild and domesticated lineages [33,57]. However, *MAL* expansion can be viewed as a domestication footprint and in fact this population has additional signatures of domestication like a type of inactivation mutation of *AQY1* and the presence of region B (Table S1) that are typical of wine strains. Consequently, China VIII must be regarded as a feral population, not a wild one.

A recent study included a comprehensive genomic survey of *S. cerevisiae* that involved 362 strains from the Wine clade [9]. Following earlier publications [6], this clade was referred to as “Wine/European” in spite of the well-recognized global distribution of wine strains, not only from Europe, but also from North and South America, South Africa and Oceania, the enrichment in European strains in this clade being simply the likely consequence of an historical sampling bias in Europe. Moreover, the truly European population corresponds to a wild South European population, closely related, but phylogenetically distinct from the wine yeasts [58]. This is so far the only population to which the epithet “European” can be appropriately applied since all members of this population were found exclusively in Europe. In addition. the claim that wild strains represent 16% of the isolates of the Wine clade [9], needs to be verified since the finding of a strain exhibiting key genomic features of the Wine clade, but isolated from soil or another non-wine substrate, does not warrant the classification as wild, as feral may be a more adequate descriptor, thus avoiding confusion with truly wild strains.

Recently, the genomes of 266 wild and domesticated Asian isolates were sequenced and it was proposed that China and Far East Asia are the center of domestication of *S. cerevisiae* [33]. In another recent study, 1011 genomes were analyzed and although the “out-of-China” model for wild populations of *S. cerevisiae* was supported, it was suggested that several independent domestication events explain the emergence of domesticated populations [9]. Here, we combined key representative sequences of both studies and although most of the phylogenetic structure of *S. cerevisiae* from previous studies could be recovered, a supposedly distinctive position of Asian domesticated lineages, compatible with an initial domestication in China from which all other domestications derive, could not be confirmed. Instead, most Chinese domesticated strains appear closely related and were gathered in a new clade (Clade 12, Chinese fermentations) that is nevertheless distinct from the Sake clade (Clade 15), a key group in most population studies, but that was not adequately represented in the study of Asian strains.

By combining genome sequences obtained in recent studies by other authors and those sequenced by us, we could observe that some of the Chinese wild populations, which globally represent the most diverse assemblage of populations in the species, have members in other regions, including other continents. This illustrates not only the broad range of the distribution of some wild populations, but also their ability to migrate over thousands of kilometers, for example, across the Indian or Pacific oceans. The mechanisms and consequences of such long range dispersal still await a formal study. Another relevant aspect is the coexistence in sympatry of distinct wild lineages without apparent relevant genetic contact. Again, the drivers for such long-term sympatric coexistence of different genetic lineages remain unknown.

If our current understanding of *S. cerevisiae* populations is taken into consideration, a wide diversity of lifestyles appears as the hallmark of this species. Such diversity is still remarkable when only wild populations are considered since arboreal (low-sugar) and sap-fruit-nectar (high-sugar) lifestyles are known. This has not been observed in *S. cerevisiae* closest relative, *S. paradoxus*, in spite of their close genetic resemblance and even sympatry of these two species in certain temperate regions [59,60]. We speculate that the better adaptation of *S. cerevisiae* to higher temperatures, reflected in its biogeography [60], allowed the colonization of tropical regions and the consequent transition to high-sugar niches. In several ways, the transitions promoted by humans during the fermentation of beverages or foodstuffs, mimic the transitions in nature from low-sugar to high-sugar niches. Independent inactivation of aquaporin genes in the wild and in the winery are an indication of such plasticity.

Recently, we proposed a domestication model with transitions from a primarily domesticated state, exemplified by wine yeasts, to a secondarily domesticated state, exemplified by cachaça-bioethanol yeasts [29]. The regions A, B and C, likely acquired first by wine strains [20], are also present (especially region B) in secondarily domesticated lineages that derive from wine yeasts like Bread, Beer 2-Diastaticus and Cachaça–Bioethanol (Figure 1). In sharp contrast, these regions are absent in wild lineages.

Contrary to regions A, B and C that were acquired solely during domestication, other traits like aquaporin inactivation, the presence of the *RTM1* cluster or the presence of the *BIO1*/*BIO6* genes appear to have predated domestication. We observed that such traits already occur in wild populations, especially in Asian ones. Therefore, the presence of the *RTM1* cluster or the *BIO1*/*BIO6* genes in certain domesticated populations may originate in the wild genetic stock of the species. Likewise, the propensity for aquaporin inactivation observed in domesticated populations may recapitulate a natural phenomenon that occurs in wild populations. For *MEL* alleles and in spite of their still unclear evolutionary history, which also involves *S. paradoxus,* their origin in *S. cerevisiae* is again centered in Asia and in wild populations.

## Figures and Tables

**Table 1:** Synonyms of *Saccharomyces cerevisiae* analyzed in this study and relevant information pertaining to them (the type strain of *S. cerevisiae* is also included).

Original Name	Strain Number^#^	Strain Number (Other Collections)	Strain Status	Population	Substrate of Isolation	Locality of Isolation
*Saccharomyces aceti* Santa María 1958	CBS 4054	–	type strain	WINE-PDM	red wine	Spain
*Saccharomyces annulatus* Negroni 1929	PYCC 6727	CBS 1227	type strain	WINE-MAIN	abscess on epididymis	New Zealand
*Saccharomyces boulardii* Seguela, Bastide & Massot 1984 (invalid name)	YJS 5879	-	type strain	WINE-MAIN	lychee	Vietnam
*Saccharomyces brasiliensis* Lindner 1909; *Saccharomyces logos* van Laer ex Jörgenssen 1909	PYCC 4602	CBS 382	type strain	BEER 2- DIASTATICUS	beer (Logos brewery)	Rio de Janeiro, Brazil
*Saccharomyces capensis* van der Walt & Tscheuschner 1956	CBS 2247	-	type strain	WINE-PDM	grape must	South Africa
*Saccharomyces cerevisiae* Meyen ex Hansen 1883	PYCC 4455	CBS 1171	neotype strain	BEER 1	brewer’s top yeast	Oranjeboom brewery, Rotterdam, Netherlands
*Saccharomyces cerevisiae* Hansen var. *onychophilus* Zach 1934	CBS 1464	-	type strain	BREAD	nail of 4-year-old girl	Austria
*Saccharomyces cerevisiae* Hansen var. *orsati* Steiner 1924	CBS 1175	-	type strain	WINE-MAIN	wine	unknown
*Saccharomyces chevalieri* Guilliermond 1914	PYCC 8489	NRRL Y-12,633; CBS 400	type strain	WEST AFRICA	palm wine from *Elaeis guineensis*	Ivory Coast
*Saccharomyces chodati* Steiner 1924	CBS 423	-	type strain	WINE-MAIN	wine	Switzerland
*Saccharomyces cordubensis* Santa María 1970	CBS 6007	-	type strain	WINE-PDM	wine	Spain
*Saccharomyces coreanus* Saito 1910	CBS 5635	-	neotype strain	WINE-MAIN	grape must	South Africa
*Saccharomyces diastaticus* Andrews & Gilliland ex van der Walt 1965	PYCC 2608	CBS 1782	type strain	BEER 2-DIASTATICUS	super-attenuated beer	unknown
*Saccharomyces carlsbergensis* Hansen var. *mandschuricus* (Saito) Stelling-Dekker 1931	CBS 1508	-	type strain	SAKE	starter for sorghum brandy	unknown
*Saccharomyces ellipsoideus* Reess 1870	PYCC 4653	CBS 1395; NRRL Y-1529	neotype strain	WINE—MAIN	unknown	unknown
*Saccharomyces ellipsoideus* Hansen var. *umbra* Castelli 1933	CBS 457	-	type strain	WINE—MAIN	grape must	Italy
*Saccharomyces ellipsoideus* Hansen var. *alpinus* Steiner 1924	CBS 1192	-	type strain	WINE—MAIN	wine	unknown
*Saccharomyces ellipsoideus* Hansen var. *montibensis* Steiner 1924	CBS 1479	-	type strain	WINE—MAIN	wine	unknown
*Saccharomyces ellipsoideus* Hansen ssp. *thermophilus* Steiner 1924	CBS 1194	-	type strain	WINE—MAIN	wine	unknown
*Saccharomyces fructuum* Lodder & Kreger-van Rij 1952	PYCC 4654	CBS 1544	type strain	OUTLIER	fermenting fruit juice	Netherlands
*Saccharomyces elongatus* Krumbholz 1932	CBS 439	-	type strain	WINE—MAIN	Silvaner grapes	Germany
*Saccharomyces formosensis* Nakazawa 1933	CBS 440	-	type strain	SAKE	molasses	Taiwan
*Saccharomyces gaditensis* Santa María 1970	CBS 6006	-	type strain	WINE—PDM	wine	Spain
*Saccharomyces hienipiensis* Santa María 1962	PYCC 6733	VKM Y-1235	type strain	OLIVES	alpechin	Spain
*Saccharomyces hispalensis* Santa María 1978	PYCC 8023	CBS 7002	type strain	OLIVES	alpechin	Seville, Spain
*Saccharomyces hispanica* Santa María 1968	CBS 5835	-	type strain	WINE-PDM	wine	Spain
*Saccharomyces ilicis* Grönlund 1893	CBS 1173	-	type strain	OUTLIER	fruit of *Ilex aquifolium*	unknown
*Saccharomyces italicus* Castelli 1939	CBS 459	-	type strain	WINE—MAIN	grape must	Italy
*Saccharomyces italicus* var. *melibiosi* van Uden & Assis-Lopes 1957	PYCC 2613	CBS 2909	type strain	OLIVES	feces of man	Portugal
*Saccharomyces lindneri* Guilliermond 1914	CBS 403	PYCC 4571	type strain	WEST AFRICA	ginger beer from *Zinziber officinale*	West Africa
*Saccharomyces mangini* var. *casei* Saccchetti 1933	PYCC 6721	CBS 420; VKM Y-482	type strain	DAIRY	stracchino cheese	Italy
*Saccharomyces marchalianus* Kufferath 1920	PYCC 8196	CBS 1460	type strain	WINE—MAIN	fermenting fruit	Indonesia
*Saccharomyces multisporus* Jörgensen 1909	CBS 4507	-	type strain	WINE—MAIN	English top brewing yeast	unknown

**Table 2:** Populations of *S. cerevisiae* and their designations.

Population	Designation in Other Studies	Life Style (W-Wild; D-Domesticated; F-Feral)	Number of Type Strains of Former *Saccharomyces* Species	Comments
1–Wine-main	Wine [25]; Wine/European [6,9]	D	17	Global distribution probably associated with the widespread dissemination of winemaking
2–Olives	Alpechin [9]; Olives [26,27]	D	6	Ancestral hybrids with *S. paradoxus* associated with processed olives
3–Wine-PDM	Flor [25,28]; Georgian [9]; PDM-Prise de Mousse [12];	D	6	Lalvin EC1118, a well-known commercial strain in this group is also known as “Prise de mousse” or Champagne strain [20,28]. This population also contains the Spanish flor yeasts of Xerez wine (Sherry) and similar wines [28]
4–Cachaça-Bioethanol	Brazilian bioethanol [9]; Cachaça C1 and C2 [29]; Rum and bioethanol [25]	D	–	This group is a secondary domesticate derived from wine strains and includes two types of cachaça strains (C1 and C2) and bioethanol strains [29]
5–Beer 2-Diastaticus	Beer 2 [30]; Mosaic beer [9]	D	6	Contains Saison-type low-gravity beer strains and beer-spoilage strains with diastase activity
6–African beer	African beer [9]	D	–	Includes strains that ferment malted millet to produce bantu beer or malted sorghum to produce bili-bili or kaffir beer
7–Wild Brazil 3-French Guiana–Mexican agave	Mexican agave, French Guiana human [9]; Wild Brazil B3 [31]	W/D	–	Complex clade composed of three subclades, one containing wild strains found in Brazil, another containing strains used in artisanal mezcal fermentation in Mexico and a third one containing gut-associated strains from French Guiana aborigines; this last group contains *STA1* and was also found in cachiri, a traditional beer made from chewed and fermented starch-rich manioc [32]
8–Mediterranean oak	-	W	–	Unique wild population with no evident links to the core group of Asian wild populations; no changes in designation since the original description [8]
9–Dairy	Cheese [25]; French dairy [9]; Milk [33]	D	1	Recently revealed population associated with dairy products and adapted to galactose utilization
10–Bread	Active dry yeast [33]; Mixed [30]	D	2	
11–Beer 1	Ale beer [9]; Beer [25]; Beer 1 [30]	D	1	Various sub-populations associated with different ale-beer types
12–Chinese fermentations (bread and beverages)	Mantou (bread)/Baijiu (distilled)/Huangjiu (rice wine)/Qingkejiu (barley wine)/fermented milk [33]	D	–	Predominantly Chinese domesticated strains that are distinct from strains of the Sake clade
13–Cocoa	West African cocoa [9]	D	–	
14–China VIII	-	F	–	Found in the arboreal niche in China, but with several domestication signatures (*MAL* gene expansion, *AQY* inactivation, presence of region B)
15–Sake	Asia [27,30]; Asian fermentation, Sake [6,9]	D	3	
16–Philippines	Asian islands [9]; Philippines [27]	W/D	–	
17–North America–Japan-Far East Russia–China VI-VII	China VI-VII [33,34]; Far East Russia [9]; North American [6]	W	–	A wild Chinese population also found in North America, Japan and Russia
18–China VI-VII	-	W	–	No changes in designation since the original description [34]
19–West Africa	African palm wine [9]; West Africa [6];	D	2	
20–Wild Brazil 1–China V	Ecuadorean [9]; Wild Brazil B1 [31]	W	–	A wild Chinese population also found in Brazil and Ecuador
21–China I	-	W	–	No changes in designation since the original description [34]
22–Malaysia	-	W	–	No changes in designation since the original description [6]
23–China III	-	W	–	No changes in designation since the original description [34]
24–China IV	-	W	–	No changes in designation since the original description [34]
25–China I	-	W	–	No changes in designation since the original description [34]
26–China IX	-	W	–	No changes in designation since the original description [34]
27–China II	-	W	–	No changes in designation since the original description [34]

## Supplementary Materials

The following are available online at https://www.mdpi.com/2076-2607/8/6/903/s1, Figure S1: Unrooted phylogeny *S. cerevisiae* genomes representing the known diversity of the species, Figure S2: °Brix measurements during growth in beer wort for 15 days at 25 °C, Figure S3: Growth curves on 1% (*w*/*v*) melibiose medium supplemented with yeast nitrogen base and incubated at 25 °C for three days, Table S1: Strains and genomes used in this study and relevant information pertaining to them, Table S2. Presence (+) or absence (−) of the *MEL* gene in the genomes of *S. paradoxus* from different populations.
