# Supplementary material for: Revisiting the Taxonomic Synonyms and Populations of Saccharomyces cerevisiae—Phylogeny, Phenotypes, Ecology and Domestication
Source: Microorganisms. 2020 Jun 15;8(6):903. doi: 10.3390/microorganisms8060903 (PMC7356373; doi:10.3390/microorganisms8060903)
Supplement: Supplementary file 1 [file microorganisms-08-00903-s001.pdf]

**Table S1. Strains and genomes used in this study and relevant information pertaining to them.**

| Strain | Other designations | Original species designation (correspondent type strain) | Substrate | Geographic location | Clime Zone (wild /feral pop.) | Phylogeny | Clade | Heterozygosity[1] | Regions[2] | | | AQY1[3] | | | AQY2[3] | | | RTM1[4] | BIO1 BIO6[5] | STA1[6] | MEL[7] | | | GENOME DATA | REFERENCE |
|---|---|---|---|---|---|---|---|---|---|---|---|---|---|---|---|---|---|---|---|---|---|---|---|---|---|
| | | | | | | | | | A | B | C | | | | | | | | | | | | | | |
| CBS 1175 | | *Saccharomyces cerevisiae* var. *orsati* | Wine | Switzerland | | WINE - Main | 1 | 3522 | 15/15 | | | NF | A881del | | NF | 11bp del | | x | x | x | x | | | ERP014555 | Peter et al. 2018 |
| CBS 1194 | | *Saccharomyces ellipsoideus* var. *thermophilus* | Wine | Unknown | | WINE - Main | 1 | 1597 | | | | NF | A881del | | NF | 11bp del | | x | x | x | x | | | ERP014555 | Peter et al. 2018 |
| CBS 1479 | | *Saccharomyces ellipsoideus* var. *montibensis* | Wine | Unknown | | WINE - Main | 1 | 1083 | | | | NF | A881del | | NF | 11bp del | | x | x | x | x | | | ERP014555 | Peter et al. 2018 |
| CBS 1192 | | *Saccharomyces ellipsoideus* var. *alpinus* | Wine | Unknown | | WINE - Main | 1 | 1183 | 15/15 | | 18/19 | NF | A881del | | NF | 11bp del | | x | x | x | x | | | ERP014555 | Peter et al. 2018 |
| CBS 423 | PYCC 2607 | *Saccharomyces chodati* | Wine | Switzerland | | WINE - Main | 1 | 4813 | 15/15 | | 18/19 | NF | A881del | | NF | 11bp del | | x | x | x | x | | | ERP014555 | Peter et al. 2018 |
| CBS 436 | PYCC 8195 | *Saccharomyces yedo* | Sake moto | Japan | | WINE - Main | 1 | n.d. | | 5/5 | 18/19 | NF | A881del | | NF | 11bp del | | x | x | x | x | | | PRJEB36095 | This study |
| CBS 457 | | *Saccharomyces ellipsoideus* var. *umbra* | Grape must | Italy | | WINE - Main | 1 | 5501 | | | 18/19 | NF | A881del | | NF | 11bp del | | x | x | x | x | | | ERP014555 | Peter et al. 2018 |
| CBS 459 | | *Saccharomyces italicus* | Grape must | Italy | | WINE - Main | 1 | 1095 | | | | NF | A881del | | NF | 11bp del | | x | x | x | x | | | ERP014555 | Peter et al. 2018 |
| PYCC 6728 | CBS 1489 | *Saccharomyces pulmonalis* | Sputum of tuberculosis patient | Pavia, Italy | | WINE - Main | 1 | 1873 | | | 18/19 | NF | A881del | | NF | 11bp del | | x | x | x | x | | | PRJEB36095 | This study |
| CBS 439 | | *Saccharomyces elongatus* | Silvaner grapes | Germany | | WINE - Main | 1 | 1758 | 15/15 | | | NF | A881del | | NF | 11bp del | | x | x | x | x | | | ERP014555 | Peter et al. 2018 |
| PYCC 4653 | CBS 1395; NRRL Y-1529 | *Saccharomyces ellipsoideus* | Unknown | Unknown | | WINE - Main | 1 | 1988 | | | | NF | A881del | | NF | 11bp del | | x | x | x | x | | | PRJEB36095 | This study |
| PR | | | Pasteur Red | Unknown | | WINE - Main | 1 | 1715 | | | | NF | A881del | | NF | 11bp del | | x | x | x | x | | | PRJEB19382 | Almeida et al. 2017 |
| CBS 4507 | | *Saccharomyces multisporus* | Brewer's yeast, English top yeast | Unknown | | WINE - Main | 1 | 2995 | | | 17/19 | NF | A881del | | NF | 11bp del | | x | x | x | x | | | ERP014555 | Peter et al. 2018 |
| PYCC 6727 | CBS 1227 | *Saccharomyces annulatus* | Human skin infection | France | | WINE - Main | 1 | 1415 | 15/15 | | 18/19 | NF | A881del | | NF | 11bp del | | x | x | x | x | | | PRJEB36095 | This study |
| EXF 6719 | | | Wine must | Cuber, Slovenia | | WINE - Main | 1 | 2053 | | | 17/19 | NF | A881del | | NF | 11bp del | | ✓ | x | x | x | | | PRJEB7601 | Almeida et al. 2015 |
| ZP 641 | | | Spontaneous red wine fermentation | Castelo de Vide, Portugal | | WINE - Main | 1 | 1484 | | | | NF | A881del | | NF | 11bp del | | x | x | x | x | | | PRJEB7601 | Almeida et al. 2015 |
| Lalvin BM45 | AWRI 1486 | | Commercial yeast (white wine) | Italy | | WINE - Main | 1 | 1280 | | 5/5 | 2/19 | NF | A881del | | NF | 11bp del | | x | x | x | x | | | PRJEB19382 | Almeida et al. 2017 |
| Lalvin CY-3079 | AWRI 2078 | | Commercial yeast (red wine) | Unknown | | WINE - Main | 1 | 1974 | | 5/5 | 4/19 | NF | A881del | | NF | 11bp del | | x | x | x | x | | | PRJEB19382 | Almeida et al. 2017 |
| PYCC 8196 | CBS 1460 | *Saccharomyces marchalianus* | Fermenting fruit | Indonesia | | WINE - Main | 1 | 1855 | | | | NF | A881del | | NF | 11bp del | | x | x | x | x | | | PRJEB36095 | This study |
| YJM 1415 | NRRL Y-268 | | Wine | France | | WINE - Main | 1 | 1256 | | 5/5 | 19/19 | NF | A881del | | NF | 11bp del | | x | x | x | x | | | PRJNA189914 | Strope et al. 2015 |
| AWRI 1631 | N 96 | | Wine | Australia | | WINE - Main | 1 | n.d. | | | 8/19 | NF | A881del | | NF | 11bp del | | x | x | x | x | | | PRJNA30553 | Borneman et al. 2008 |
| AWRI 796 | Active Dry Wine Yeast | | Commercial wine yeast | South Africa | | WINE - Main | 1 | n.d. | | DUP | | F | | | NF | 11bp del | | x | x | x | ✓ | NF | EUR | PRJNA48559 | Borneman et al. 2011 |
| CBS 5635 | | *Saccharomyces coreanus* | Grape must | South Africa | | WINE - Main | 1 | 1552 | | | | NF | A881del | | NF | 11bp del | | x | x | x | ✓ | F | EUR | ERP014555 | Peter et al. 2018 |
| YJS 5879 | ULG84F88I90 | *Saccharomyces boulardii* | Lychee (reference strain Biocodex) | Vietnam | | WINE - Main | 1 | 3918 | | 5/5 | | NF | A881del | | NF | 11bp del | | x | x | x | x | | | ERP014555 | Peter et al. 2018 |
| YJS 5881 | perentol | | Perenterol capsule | Brussels, Belgium | | WINE - Main | 1 | 3948 | | 5/5 | | NF | A881del | | NF | 11bp del | | x | x | x | x | | | ERP014555 | Peter et al. 2018 |
| YJS 5880 | ultra lev. 1127 | | UltraLevure packet | Paris, France | | WINE - Main | 1 | 3956 | | 5/5 | | NF | A881del | | NF | 11bp del | | x | x | x | x | | | ERP014555 | Peter et al. 2018 |
| VL3 | B6, AWRI 1688 | | Commercial wine yeast | Laffort | | WINE - Main | 1 | n.d. | | 5/5 | 8/19 | NF | A881del | | NF | 11bp del | | x | x | x | x | | | PRJNA48565 | Borneman et al. 2011 |
| A-14 | | | Wine conserved in amphora | Georgia | | WINE - Main | 1 | 9361 | | 5/5 | 2/19 | NF | A881del | | NF | 11bp del | | x | x | x | x | | | ERP014555 | Peter et al. 2018 |
| A-16 | | | Wine conserved in amphora | Georgia | | WINE - Main | 1 | 12369 | | 5/5 | 2/19 | NF | A881del | | NF | 11bp del | | x | x | x | x | | | ERP014555 | Peter et al. 2018 |
| A-19 | | | Wine conserved in amphora | Georgia | | WINE - Main | 1 | 13819 | | 5/5 | 2/19 | NF | A881del | | NF | 11bp del | | x | x | x | x | | | ERP014555 | Peter et al. 2018 |
| D-4 | | | Wine conserved in amphora | Georgia | | WINE - Main | 1 | 14899 | | 5/5 | 2/19 | NF | A881del | | NF | 11bp del | | x | x | x | x | | | ERP014555 | Peter et al. 2018 |
| CBS 5112 | | *Saccharomyces onubensis* | Grape must | Spain | | WINE - Main | 1 | 1284 | | | | NF | A881del | | NF | 11bp del | | x | x | x | x | | | ERP014555 | Peter et al. 2018 |
| PYCC 2613 | CBS 2909; NRRL YB-6041 | *Saccharomyces italicus var. melibiosi* | Faeces of man | Portugal | | OLIVES | 2 | 6783 | | | | NF | A881del | | NF | 11bp del | | x | x | x | ✓ | F | EUR | PRJEB30431 | Pontes et al. 2019 |
| CBS 7002 | PYCC 8023 | *Saccharomyces hispalensis* | Alpechin | Sevilla, Spain | | OLIVES | 2 | 2471 | | | | NF | A881del | | NF | 11bp del | | x | x | x | ✓ | F | EUR | PRJEB30431 | Pontes et al. 2019 |
| PYCC 6730 | VKM Y-1231 | *Saccharomyces oleaceus* | Olives | Spain | | OLIVES | 2 | 2965 | | 5/5 | | NF | A881del | | NF | 11bp del | | x | x | x | ✓ | F | EUR | PRJEB30431 | Pontes et al. 2019 |
| PYCC 6732 | VKM Y-1234 | *Saccharomyces oleaginosus* | Alpechin | Spain | | OLIVES | 2 | 1886 | | | | NF | A881del | | NF | 11bp del | | x | x | x | ✓ | F | EUR | PRJEB30431 | Pontes et al. 2019 |
| PYCC 6731 | VKM Y-1232 | *Saccharomyces norbensis* | Alpechin | Spain | | OLIVES | 2 | 1393 | | 4/5 | | NF | A881del | | NF | 11bp del | | x | x | x | ✓ | F | EUR | PRJEB30431 | Pontes et al. 2019 |
| PYCC 6733 | VKM Y-1235 | *Saccharomyces hienipiensis* | Alpechin | Spain | | OLIVES | 2 | 5258 | | | | NF | A881del | | NF | 11bp del | | x | x | x | ✓ | F | EUR | PRJEB30431 | Pontes et al. 2019 |
| Lalvin QA23 | | | Commercial wine yeast | Portugal | | WINE - PDM | 3 | n.d. | 15/15 | TRIP | 19/19 | NF | A881del | | F | | | x | x | x | x | | | PRJNA48561 | Borneman et al. 2011 |
| PYCC 4074 | | | Commercial wine yeast (Fermichamp) | Portugal | | WINE - PDM | 3 | 2263 | 15/15 | 5/5 | | NF | A881del | | F | | | x | x | x | x | | | PRJEB19382 | Almeida et al. 2017 |
| EC 1118 | Prise de Mousse | | Industrial strain isolated from Champagne | France | | WINE - PDM | 3 | n.d. | 15/15 | 5/5 | 19/19 | NF | A881del | | NF | 11bp del | | x | x | x | x | | | PRJEA37863 | Novo et al. 2009 |
| PYCC 6722 | CBS 5155; VKM Y-515 | *Saccharomyces prostoserdovii* | Wine | South Armenia | | WINE - PDM | 3 | 2121 | | | | NF | A881del | | F | | | x | x | x | x | | | PRJEB19382 | Almeida et al. 2017 |
| PYCC 6729 | | | Jerez-wine | Armenia | | WINE - PDM | 3 | 2901 | | | | NF | A881del | | F | | | x | x | x | x | | | PRJEB19382 | Almeida et al. 2017 |
| 5-18 | | | Wine conserved in amphora | Georgia | | WINE - PDM | 3 | 4034 | | 5/5 | | NF | A881del | | F | | | x | x | x | x | | | ERP014555 | Peter et al. 2018 |
| B-24 | | | Wine conserved in amphora | Georgia | | WINE - PDM | 3 | 5115 | | 5/5 | 19/19 | NF | A881del | | F | | | x | x | x | x | | | ERP014555 | Peter et al. 2018 |
| CBS 2247 | | *Saccharomyces capensis* | Wine | South Africa | | WINE - PDM | 3 | 1547 | | | | NF | A881del | | F | | | x | x | x | x | | | ERP014555 | Peter et al. 2018 |
| CBS 4054 | | *Saccharomyces aceti* | Wine | Spain | | WINE - PDM | 3 | 1712 | | | | NF | A881del | | F | | | x | x | x | x | | | ERP014555 | Peter et al. 2018 |
| CBS 5835 | | *Saccharomyces hispanica* | Wine | Spain | | WINE - PDM | 3 | 3825 | | | | NF | A881del | | F | | | x | x | x | x | | | ERP014555 | Peter et al. 2018 |
| CBS 6006 | | *Saccharomyces gaditensis* | Wine | Spain | | WINE - PDM | 3 | 3867 | | 5/5 | 19/19 | NF | A881del | | F | | | x | x | x | x | | | ERP014555 | Peter et al. 2018 |
| CBS 6007 | | *Saccharomyces cordubensis* | Wine | Spain | | WINE - PDM | 3 | 1520 | | | | NF | A881del | | F | | | x | x | x | x | | | ERP014555 | Peter et al. 2018 |
| PYCC 6726 | | | Jerez-wine | Spain | | WINE - PDM | 3 | 4939 | | | | NF | A881del | | F | | | x | x | x | x | | | PRJEB19382 | Almeida et al. 2017 |
| CLIB 1082 | | | Cider brewery, dry cider | Upper Normandy, France | | CACHAÇA- BIOETHANOL | 4 | 22616 | 15/15 | 5/5 | | NF | A881del | V121M | F | | | ✓ | ✓ | x | x | | | ERP014555 | Peter et al. 2018 |
| UFMG-CM-Y030 | CAY1007 | | Cachaça (commercial strain) | Minas Gerais, Brazil | | CACHAÇA- BIOETHANOL | 4 | 31074 | | 5/5 | | NF | A881del | | NF | | G25del | x | x | x | ✓ | F | SA | PRJEB24932 | Barbosa et al. 2018 |
| UFMG-CM-Y630 | SC3 | | Cachaça | Santa Catarina, Brazil | | CACHAÇA- BIOETHANOL | 4 | 3570 | | 5/5 | | F | | | NF | 11bp del | | x | ✓ | x | x | | | PRJEB24932 | Barbosa et al. 2018 |
| CBS 7959 | | | Factory producing ethanol (sugar cane syrup) | Sao Paulo, Brazil | | CACHAÇA- BIOETHANOL | 4 | 2365 | | 5/5 | | NF | A881del | | NF | 11bp del | | ✓ | ✓ | x | x | | | ERP014555 | Peter et al. 2018 |
| UFMG-CM-Y633 | T09 | | Cachaça | Tocantins, Brazil | | CACHAÇA- BIOETHANOL | 4 | 6833 | | | | NF | A881del | | F | | | ✓ | x | x | x | | | PRJEB24932 | Barbosa et al. 2018 |
| UFMG-CM-Y634 | TOC 1301 | | Cachaça | Tocantins, Brazil | | CACHAÇA- BIOETHANOL | 4 | 21251 | | 5/5 | | NF | A881del | | NF | 11bp del | | ✓ | x | x | x | | | PRJEB24932 | Barbosa et al. 2018 |
| BG-1 | | | Bioethanol-producing strain from sugar cane | Brazil | | CACHAÇA- BIOETHANOL | 4 | n.d. | | 4/5 | | NF | A881del | | NF | | 20bp ins | x | x | x | x | | | PRJNA352845 | Coutouné 2017 |
| M.9.1 | | | Cachaça | Brazil | | CACHAÇA- BIOETHANOL | 4 | 26964 | | 5/5 | | NF | | V121M | NF | 11bp del | | x | ✓ | x | x | | | ERP014555 | Peter et al. 2018 |
| M1.1 | | | Cachaça | Brazil | | CACHAÇA- BIOETHANOL | 4 | 23740 | | 5/5 | | NF | A881del | | NF | 11bp del | | x | ✓ | x | x | | | ERP014555 | Peter et al. 2018 |
| BE063 | | | Beer | England | | BEER 2 | 5 | n.d. | 15/15 | 5/5 | | NF | | V121M | F | | | ✓ | x | x | x | | | MBXR00000000 | Gallone et al. 2016 |
| CBS 1398 | | *Saccharomyces thermantitonum* | Leaf of *Eucalyptus* sp. | Unknown | | BEER 2 | 5 | 16595 | 15/15 | 5/5 | | NF | | V121M | NF | 11bp del | | ✓ | x | x | x | | | ERP014555 | Peter et al. 2018 |
| CBS 381 | PYCC 4566 | *Saccharomyces willianus* | Spoiled beer | Japan | | BEER 2 | 5 | 12832 | | | | NF | | V121M | NF | | G25del | ✓ | x | x | x | | | PRJEB36095 | This study |
| CBS 422 | PYCC 4572 | *Saccharomyces odessa* | Beer | Ukraine | | BEER 2 | 5 | 1771 | 15/15 | | | NF | A881del | V121M | NF | 11bp del | | ✓ | x | x | x | | | ERP014555 | Peter et al. 2018 |
| PYCC 4602 | CBS 382 | *Saccharomyces brasiliensis* | Logos brewer's | Rio de Janeiro, Brazil | | BEER 2 | 5 | 20999 | 15/15 | 5/5 | | NF | A881del | | NF | 11bp del | G25del | ✓ | x | x | ✓ | F | AA | PRJEB36095 | This study |
| CBS 1173 | | *Saccharomyces ilicis* | Fruit of *Ilex aquifolium* | Unknown | | BEER 2 | 5 | 12355 | | | | NF | A822ins | V121M | NF | 11bp del | | ✓ | x | x | x | | | ERP014555 | Peter et al. 2018 |
| CBS 2444 | PYCC 4574 | | Seagrams distillery | USA | | BEER 2 | 5 | 41509 | | | | NF | A881del | V121M | NF | 11bp del | | ✓ | x | x | ✓ | F | AA | PRJEB36095 | This study |
| BE092 | | | Beer (Strong ale) | Belgium | | BEER 2 | 5 | n.d. | 15/15 | | | NF | A881del | | F | | | ✓ | x | ✓ | x | | | MBWO00000000 | Gallone et al. 2016 |
| TUM 71 | | | Spoilage yeast Isolated from brewery | Germany, State North-Rhine Westphalia (Nordrhein-Westfalen) | | BEER 2 | 5 | 20555 | 15/15 | 5/5 | | NF | | V121M | NF | 11bp del | | ✓ | x | ✓ | ✓ | F | AF I | PRJEB36095 | This study |
| BE013 | | | Beer (Ale refermentation) | Belgium | | BEER 2 | 5 | n.d. | | 5/5 | | NF | A881del | | F | | | ✓ | ✓ | ✓ | x | | | MBZP00000000 | Gallone et al. 2016 |
| BE083 | | | Beer (Saison) | Belgium | | BEER 2 | 5 | n.d. | | 5/5 | | NF | A881del | | NF | 11bp del | | ✓ | ✓ | ✓ | x | | | MBWX00000000 | Gallone et al. 2016 |
| TUM PI-BA-45 | | | Spoilage yeast Isolated from brewery | Germany, State Lower Saxony (Niedersachsen) | | BEER 2 | 5 | 25787 | | 5/5 | | NF | | V121M | NF | | 786 G>A stop | ✓ | ✓ | ✓ | x | | | PRJEB36095 | This study |
| TUM PI-BA-109 | | | Spoilage yeast Isolated from brewery | Germany, State North-Rhine Westphalia (Nordrhein-Westfalen) | | BEER 2 | 5 | 21268 | | 5/5 | | NF | A881del | | NF | 11bp del | | ✓ | x | ✓ | x | | | PRJEB36095 | This study |
| TUM PI-BA-31 | | | Spoilage yeast Isolated from brewery | Germany, State Rhineland-Palatinate (Rheinland-Pfalz) | | BEER 2 | 5 | 27650 | | DUP | | NF | | V121M | NF | 11bp del | | ✓ | x | ✓ | x | | | PRJEB36095 | This study |
| TUM PI-BB-105 | | | Spoilage yeast Isolated from beer-mixed beverage | Unknown | | BEER 2 | 5 | 23206 | 15/15 | 5/5 | | NF | A881del | | NF | 11bp del | | ✓ | ✓ | ✓ | x | | | PRJEB36095 | This study |
| TUM 3-D-2 | | | Spoilage yeast Isolated from brewery | Germany, State Lower Saxony (Niedersachsen) | | BEER 2 | 5 | 28706 | | 5/5 | | NF | A881del | | F | | | ✓ | x | ✓ | x | | | PRJEB36095 | This study |
| TUM 3-H-2 | | | Spoilage yeast Isolated from brewery | Germany, State Lower Saxony (Niedersachsen) | | BEER 2 | 5 | 28485 | | 5/5 | | NF | A881del | | F | | | ✓ | x | ✓ | x | | | PRJEB36095 | This study |

| | | | | | | | | | | | | | | | | | | | | | | | | | |
|---|---|---|---|---|---|---|---|---|---|---|---|---|---|---|---|---|---|---|---|---|---|---|---|---|---|
| BE021 | | | Beer (Pale ale) | Canada | | BEER 2 | 5 | n.d. | | 5/5 | | NF | A881del | | F | | | ✓ | x | ✓ | x | | | MBZH00000000 | Gallone et al. 2016 |
| BE032 | | | Beer | England | | BEER 2 | 5 | n.d. | | 5/5 | 4/19 | NF | A881del | | NF | 11bp del | | ✓ | x | ✓ | x | | | MBYW00000000 | Gallone et al. 2016 |
| CBS 6505 | | | Brewery (killer strain responsible for death of brewing yeast) | UK | | BEER 2 | 5 | 2212 | | 5/5 | | NF | A881del | | NF | 11bp del | | ✓ | x | ✓ | x | | | ERP014555 | Peter et al. 2018 |
| CBS 7958 | | | Factory producing cassava flour | Sao Paulo, Brazil | | BEER 2 | 5 | 2183 | | 5/5 | 19/19 | NF | A881del | | F | | | x | x | ✓ | x | | | ERP014555 | Peter et al. 2018 |
| PYCC 2608 | CBS 1782 | *Saccharomyces diastaticus* | Super-attenuated beer | Unknown | | BEER 2 | 5 | 2770 | | 5/5 | 19/19 | NF | A881del | | F | | | x | x | ✓ | x | | | PRJEB36095 | This study |
| TUM 1-B-8 | | | Spoilage yeast isolated from brewery | Germany, State Bavaria (Bayern) | | BEER 2 | 5 | 4543 | | 5/5 | 4/19 | NF | A881del | | F | | | x | x | x | x | | | PRJEB36095 | This study |
| CH14 | | | Pearl millet beer | Abengourou, Ivory Coast | | AFRICAN BEER | 6 | 27976 | | 5/5 | | F | | | NF | | 424 C>T Stop | x | x | x | x | | | ERP014555 | Peter et al. 2018 |
| CLIB 651 | | | Beer leaven for Bili Bili beer, brewery | Chad | | AFRICAN BEER | 6 | 16287 | | | | F | | | NF | | 424 C>T Stop | x | x | x | x | | | ERP014555 | Peter et al. 2018 |
| MAJ_G | | | Plant material | Majunga, Madagascar | | AFRICAN BEER | 6 | 7094 | | | | F | | | NF | | GT158ins | ✓ | x | x | ✓ | F | AF I | ERP014555 | Peter et al. 2018 |
| N26-1-10:7-5(a) | | | Maize dough | Ghana | | AFRICAN BEER | 6 | 3781 | | | | F | | | NF | | 424 C>T Stop | x | x | x | ✓ | F | AF I | ERP014555 | Peter et al. 2018 |
| PYCC 8031 | NRRL Y-7184 | | Bantu beer | van der Walt, Pretoria, South Africa | | AFRICAN BEER | 6 | 50093 | | | 5/19 | NF | | 321 G>A Stop | NF | G25del | | x | ✓ | x | ✓ | F | EUR | PRJEB36095 | This study |
| CBS 4454 | | | Kaffir beer | South Africa | | AFRICAN BEER | 6 | 17328 | | | | NF | | T498del | NF | 11bp del | | x | x | x | x | | | ERP014555 | Peter et al. 2018 |
| CBS 4456 | | | Kaffir beer | South Africa | | AFRICAN BEER | 6 | 40939 | | | 5/19 | NF | | T498del | NF | 11bp del | | x | x | x | x | | | PRJEB36095 | This study |
| UFMG-CM-Y263 | C41 | | *Tapirira guianensis* | Tocantins, Brazil | TROP | WB3 - FG -MA | 7 | 1862 | | | | F | | | F | | | x | ✓ | x | ✓ | F | SA | PRJEB11698 | Barbosa et al. 2016 |
| NRRL YB-908 | | | Wild cherry tree gum | Wickerham, NRRL GM | TROP | WB3 - FG -MA | 7 | 2715 | | | | F | | | F | | | x | ✓ | x | x | | | PRJEB36095 | This study |
| UFMG-CM-Y641 | TOC 1345 | | Tapirira guianensis | Tocantins, Brazil | TROP | WB3 - FG -MA | 7 | 2543 | | | | F | | | F | | | x | ✓ | x | ✓ | F | SA | PRJEB11698 | Barbosa et al. 2016 |
| UFMG-CM-Y642 | TOC 1365 | | Tapirira guianensis | Tocantins, Brazil | TROP | WB3 - FG -MA | 7 | 1862 | | | | F | | | F | | | x | ✓ | x | ✓ | F | SA | PRJEB11698 | Barbosa et al. 2016 |
| CEY 619 | | | Human feces | French Guiana | TROP | WB3 - FG -MA | 7 | 10245 | | 5/5 | | NF | | T239 del | F | | | x | ✓ | ✓ | x | | | ERP014555 | Peter et al. 2018 |
| HE 004 | | | Human feces | French Guiana | TROP | WB3 - FG -MA | 7 | 11945 | | 5/5 | | NF | A822ins | T239 del | F | | | x | ✓ | ✓ | x | | | ERP014555 | Peter et al. 2018 |
| HE 012 | | | Human feces | French Guiana | TROP | WB3 - FG -MA | 7 | 9103 | | 5/5 | | NF | A822ins | T239 del | F | | | x | ✓ | ✓ | x | | | ERP014555 | Peter et al. 2018 |
| LCBG-3D6 | | | *Agave* spp. fermentation during rustic mezcal production | Tamaulipas, Mexico | TROP | WB3 - FG -MA | 7 | 17358 | | 5/5 | | NF | | T230 del | F | | | ✓ | ✓ | x | x | | | ERP014555 | Peter et al. 2018 |
| LCBG-3Y8 | | | *Agave* spp. fermentation during rustic mezcal production | Tamaulipas, Mexico | TROP | WB3 - FG -MA | 7 | 22105 | | 5/5 | | NF | | T230 del | F | PAR NA | | ✓ | ✓ | x | x | | | ERP014555 | Peter et al. 2018 |
| LCBG-3D2 | | | *Agave* spp. fermentation during rustic mezcal production | Tamaulipas, Mexico | TROP | WB3 - FG -MA | 7 | 17358 | | 5/5 | | NF | | T230 del | NF | | G107 del | ✓ | ✓ | x | x | | | ERP014555 | Peter et al. 2018 |
| MB 7c | | | *Quercus pubescens* | Montbarri, Southern France | TEMP | MO | 8 | 3752 | | | | F | | | F | | | x | x | x | x | | | PRJNA264372 | Almeida et al. 2015 |
| ZP 560 | | | *Quercus pyrenaica* | Castelo de Vide, Portugal | TEMP | MO | 8 | 2553 | | | | F | | | F | | | x | x | x | x | | | PRJEB7601 | Almeida et al. 2015 |
| ZP 848 | | | *Quercus ilex* | Alter do Chão, Portugal | TEMP | MO | 8 | 2656 | | | | F | | | F | | | x | x | x | x | | | PRJEB7675 | Almeida et al. 2015 |
| ZP 570 | | | *Fraxinus* sp. | Paul Boquilobo, Portugal | TEMP | MO | 8 | 2813 | | | | F | | | F | | | x | x | x | x | | | PRJEB7601 | Almeida et al. 2015 |
| ZP 850 | | | *Quercus ilex* | Alconorales Natural Park, Andaluzia, Spain | TEMP | MO | 8 | 2869 | | | | F | | | F | | | x | x | x | x | | | PRJEB7601 | Almeida et al. 2015 |
| ZP 541 | | | *Fagus sylvatica* | Adagoi, Portugal | TEMP | MO | 8 | 2684 | | | | F | | | F | | | x | x | x | x | | | PRJEB7601 | Almeida et al. 2015 |
| 3.10 | DBVPG 10100 | | *Quercus cerris* | Parco del Monte Subasio, Italy | TEMP | MO | 8 | 3800 | | | | F | | | F | | | x | x | x | x | | | PRJEB7601 | Almeida et al. 2015 |
| DBVPG 10158 | | | soil underneath *Quercus cerris* | Riserva Naturale Lucciolabella/Chianciano (SI) 2012 | TEMP | MO | 8 | n.d. | | | | F | | | F | | | x | x | x | x | | | PRJEB36095 | This study |
| HUN 9.1s1 | DBS 14 | | Oak | Hungary | TEMP | MO | 8 | 2745 | | | | F | | | F | | | x | x | x | x | | | PRJEB7601 | Almeida et al. 2015 |
| EXF 7200 | | | *Quercus robur* | Jasenovo polje, Montenegro | TEMP | MO | 8 | 3001 | | | | F | | | F | | | x | x | x | x | | | PRJNA264372 | Almeida et al. 2015 |
| OakRom 3-2a | | | Oak | Near Bucarest, Romania | TEMP | MO | 8 | 2366 | | | | F | | | F | | | x | x | x | x | | | PRJEB7675 | Almeida et al. 2015 |
| PYR 4b | DBQ 26 | | *Quercus pubescens* | Halkidiki, Greece | TEMP | MO | 8 | 2738 | | | | F | | | F | | | x | x | x | x | | | PRJEB7601 | Almeida et al. 2015 |
| CBS 2421 | PYCC 2503 | | Japanese kefyr grains | Unknown | | DAIRY | 9 | 27903 | 15/15 | 5/5 | | NF | A881del | | NF | 11bp del | | x | x | x | x | | | ERP014555 | Peter et al. 2018 |
| VKMY 373 | | | Camembert cheese | France | | DAIRY | 9 | n.d. | | | | NF | A881del | | NF | 11bp del | | x | x | x | x | | | PRJEB7675 | Legras et al. 2018 |
| KS11 | | | Kumiss | Kazakhistan | | DAIRY | 9 | n.d. | | | | NF | A881del | | NF | 11bp del | | ✓ | x | x | x | | | PRJEB7675 | Legras et al. 2018 |
| PYCC 6721 | CBS 420; VKM Y-482 | *Saccharomyces mangini* | Cheese | Italy | | DAIRY | 9 | 37556 | | | | NF | A881del | 88 C>T Stop | NF | 11bp del | | x | x | x | x | | | PRJEB36095 | This study |
| PYCC 6720 | CBS 437, NRRL Y-1545 | | Stracchino Cheese | Italy | | DAIRY | 9 | 23692 | | | | NF | V121M | | NF | | 185 T>A Stop | x | x | x | x | | | PRJEB36095 | This study |
| EXF 5871 | | | Homemade cheese | Slovenia | | DAIRY | 9 | 27590 | | | | NF | A881del | | NF | | 185 T>A Stop | x | x | x | x | | | ERP014555 | Peter et al. 2018 |
| CLIB 554 | | | Dairy, brine | France | | DAIRY | 9 | 24196 | | | | NF | A881del | | NF | 11bp del | | x | x | x | x | | | ERP014555 | Peter et al. 2018 |
| CLIB 553 | | | Dairy, raw milk | Normandy, France | | DAIRY | 9 | 10209 | | | | NF | A881del | | NF | | 185 T>A Stop | x | x | x | x | | | ERP014555 | Peter et al. 2018 |
| MUCL 42908 | | | Kefir | Morrocco | | DAIRY | 9 | n.d. | | | | NF | A881del | | NF | | 185 T>A Stop | x | x | x | x | | | PRJEB7675 | Legras et al. 2018 |
| Morocco Bread G17 | MBG17 | | Baker's yeast | | | BREAD | 10 | 61910 | | 5/5 | | NF | V121M | A822ins | F | | | ✓ | x | x | x | | | SRR403240 | Dunn et al. 2012 |
| PYCC 5324 | | | Homemade corn and rye bread dough | Braga, Portugal | | BREAD | 10 | 53843 | | 5/5 | | NF | V121M | A822ins | NF | 11bp del | | ✓ | x | x | x | | | PRJEB36095 | This study |
| SD-15 | | | Sourdough levan | Matera, Italy | | BREAD | 10 | 50578 | | 5/5 | | NF | V121M | A822ins | F | | | ✓ | x | x | x | | | ERP014555 | Peter et al. 2018 |
| PYCC 5320 | | | Homemade corn and rye bread dough | Braga, Portugal | | BREAD | 10 | 54495 | | 4/5 | | NF | V121M | | NF | 11bp del | | ✓ | x | x | x | | | PRJEB36095 | This study |
| AP13.1 | | | Fermented sourdough | Cinfães, Portugal | | BREAD | 10 | 53091 | | 5/5 | 17/19 | NF | V121M | | NF | 11bp del | | ✓ | x | x | x | | | PRJEB24932 | Barbosa et al. 2018 |
| BR004 | | | Bread | Belgium | | BREAD | 10 | n.d. | | 5/5 | 17/19 | NF | V121M | | NF | 11bp del | | ✓ | x | x | x | | | MBVV00000000 | Gallone et al. 2016 |
| CBS 2361 | | | Trinidadian cane sugar | UK | | BREAD | 10 | 37044 | | 5/5 | 17/19 | NF | A881del | | NF | | 786 G>A stop | ✓ | x | x | x | | | PRJEB36095 | This study |
| CBS 1464 | | *Saccharomyces cerevisiae var. onychophilus* | Infected nail of 4 year old girl | Austria | | BREAD | 10 | 2407 | | | | NF | V121M | A822ins | NF | 11bp del | | ✓ | x | x | x | | | ERP014555 | Peter et al. 2018 |
| NRRL YB-3916 | CBS 2919 | *Saccharomyces. veronae var. osloensis* | Sputum | Oslo, Norway | | BREAD | 10 | 44584 | | 5/5 | | NF | A881del | | NF | 11bp del | | ✓ | x | x | x | | | PRJEB36095 | This study |
| PYCC 4226 | | | Commercial baker's yeast | Unknown | | BREAD | 10 | 30081 | | 4/5 | 2/19 | NF | A881del | | NF | | 792stop | ✓ | x | x | x | | | ERS1108635 | Gonçalves et al. 2016 |
| Platinum | | | Commercial baker's yeast | Unknown | | BREAD | 10 | 42278 | | 4/5 | 15/19 | NF | V121M | A822ins | NF | | 792stop | ✓ | x | x | x | | | ERS1108633 | Gonçalves et al. 2016 |
| TUM 308 | | | Alt beer | Rhineland-Palatinate, Germany | | BEER 1 | 11 | 33689 | | | | NF | V121M | | NF | 11bp del | 792stop | ✓ | x | x | x | | | ERS1108619 | Gonçalves et al. 2016 |
| TUM 513 | | | California ale | Unknown | | BEER 1 | 11 | 33061 | | | | NF | V121M | | NF | 11bp del | 792stop | ✓ | x | x | x | | | ERS1108618 | Gonçalves et al. 2016 |
| TUM 174 | | | Regional beer similar to Kölsch beer | Mülheim, Germany | | BEER 1 | 11 | 21227 | | | | NF | V121M | | NF | 11bp del | 792stop | ✓ | x | x | x | | | ERS1108621 | Gonçalves et al. 2016 |
| PYCC 4455 | CBS 1171; NRRL Y-12632 | *Saccharomyces cerevisiae* | Brewer's top yeast | Oranjeboom brewery, Rotterdam, Netherlands | | BEER 1 | 11 | 22852 | | | | NF | V121M | | NF | 11bp del | | ✓ | x | x | x | | | PRJEB36095 | This study |
| TUM 205 | | | Wheat Beer | | | BEER 1 | 11 | 41573 | | | | NF | V121M | | NF | 11bp del | | ✓ | x | x | x | | | ERS1108614 | Gonçalves et al. 2016 |
| BE074 | | | Beer (Hefeweizen) | Germany | | BEER 1 | 11 | n.d. | | | | NF | V121M | | F | | | ✓ | x | x | x | | | MBXG00000000 | Gallone et al. 2016 |
| TUM 175 | | | Wheat beer | Freising-Weihenstephan, Germany | | BEER 1 | 11 | 29902 | | | | NF | V121M | | F | | | ✓ | x | x | x | | | ERS1108615 | Gonçalves et al. 2016 |
| TUM 507 | | | Ale Beer from wheatmalt | Unknown | | BEER 1 | 11 | 40936 | | | | NF | V121M | | F | | | ✓ | x | x | x | | | ERS1108616 | Gonçalves et al. 2016 |
| TUM 503 | | | California ale | Unknown | | BEER 1 | 11 | 30324 | | | | NF | V121M | A822ins | NF | 11bp del | | ✓ | x | x | x | | | ERS1108626 | Gonçalves et al. 2016 |
| FostersO | | | Commercial brewing ale strain | | | BEER 1 | 11 | 34874 | | | | NF | V121M | A822ins | NF | 11bp del | | ✓ | x | x | x | | | ERS1108627 | Gonçalves et al. 2016 |
| FostersB | | | Commercial brewing ale strain | | | BEER 1 | 11 | 43000 | | | | NF | V121M | A822ins | NF | 11bp del | | ✓ | x | x | x | | | ERS1108629 | Gonçalves et al. 2016 |
| TUM 211 | | | British ale / stout | Great Britain | | BEER 1 | 11 | 26842 | | | | NF | V121M | A822ins | NF | | 792stop | ✓ | x | x | x | | | ERS1108632 | Gonçalves et al. 2016 |
| LM1-2 | | | Fermented yak milk | Naqu, Tibet | | CHN FERMENTATION | 12 | 7132 | | | | NF | A881del | | NF | | 185 T>A Stop | x | x | x | x | | | PRJNA396809 | Duan et al. 2018 |
| WM11-3 | | | Fermented mixed goat and yak milk | Mongolia | | CHN FERMENTATION | 12 | 7118 | | | | NF | A881del | 88 C>T Stop | NF | 11bp del | | x | x | x | x | | | PRJNA396809 | Duan et al. 2018 |
| 5-3004 | | | Fermented mare's milk (kumis) | Ulaanbaatar, Mongolia | | CHN FERMENTATION | 12 | 4978 | | | | NF | A881del | | NF | 11bp del | | x | x | x | x | | | PRJNA396809 | Duan et al. 2018 |
| QH21-3 | | | Fermented yak milk | Delingha, Qinghai | | CHN FERMENTATION | 12 | 7574 | | | | NF | V121M | | NF | 11bp del | | ✓ | x | x | x | | | PRJNA396809 | Duan et al. 2018 |
| JM13.2 | | | Fermenting dough | Yuncheng, Shanxi | | CHN FERMENTATION | 12 | 10349 | | | | NF | V121M | | NF | G25del | | ✓ | ✓ | x | x | | | PRJNA396809 | Duan et al. 2018 |
| WL1 | | | Fermenting dough | Wulanchabu, Inner Mongolia | | CHN FERMENTATION | 12 | 10255 | | | | NF | V121M | | NF | G25del | | ✓ | ✓ | x | x | | | PRJNA396809 | Duan et al. 2018 |
| BT2.8 | | | Fermenting dough | Baotou, Inner Mongolia | | CHN FERMENTATION | 12 | 9069 | | | | NF | V121M | | NF | G25del | | ✓ | x | x | ✓ | F | AS I | PRJNA396809 | Duan et al. 2018 |
| BT3 | | | Fermenting dough | Baotou, Inner Mongolia | | CHN FERMENTATION | 12 | 7761 | | | | NF | V121M | | F | | | ✓ | x | x | ✓ | F | R | PRJNA396809 | Duan et al. 2018 |
| QH6.5 | | | Fermenting dough | Minhe, Qinghai | | CHN FERMENTATION | 12 | 9259 | | | | NF | V121M | | n.d. | | | x | ✓ | x | x | | | PRJNA396809 | Duan et al. 2018 |
| CJM19.5 | | | Fermenting dough | Yuncheng, Shandong | | CHN FERMENTATION | 12 | 10592 | | | | NF | V121M | | NF | G25del | | x | ✓ | x | x | | | PRJNA396809 | Duan et al. 2018 |
| QH1.1 | | | Fermenting dough | Qinghai | | CHN FERMENTATION | 12 | 3753 | | | | NF | V121M | | F | | | x | ✓ | x | x | | | PRJNA396809 | Duan et al. 2018 |
| XZ2.1 | | | Daqu (starter) of Qingkejiu (highland barley wine) | Lasa, Tibet | | CHN FERMENTATION | 12 | 3232 | | | | NF | V121M | | NF | G25del | | x | ✓ | x | x | | | PRJNA396809 | Duan et al. 2018 |
| JQ9.4 | | | Fermenting dough | Nanyang, Henan | | CHN FERMENTATION | 12 | 7371 | | | | NF | V121M | | NF | G25del | | ✓ | ✓ | x | x | | | PRJNA396809 | Duan et al. 2018 |

| | | | | | | | | | | | | | | | | | | | | | | | | | | |
|---|---|---|---|---|---|---|---|---|---|---|---|---|---|---|---|---|---|---|---|---|---|---|---|---|---|---|
| CQJZ1.4 | | | Fermented rice | Shaoxing, Zhejiang | | CHN FERMENTATION | 12 | 540 | | | | NF | V121M | A822ins | NF | G25del | | x | ✓ | x | x | | | | PRJNA396809 | Duan et al. 2018 |
| HQ1.1 | | | Huangjiu (rice wine) starter with fermented monascus | Gutian, Fujian | | CHN FERMENTATION | 12 | 3753 | | | | NF | V121M | A863del | NF | G25del | | ✓ | ✓ | x | ✓ | F | AS I | | PRJNA396809 | Duan et al. 2018 |
| GZLJ3.19 | | | Fermented sorghum grain | Changshun, Guizhou | | CHN FERMENTATION | 12 | 3807 | | | | NF | V121M | A822ins | NF | G25del | | x | ✓ | x | x | | | | PRJNA396809 | Duan et al. 2018 |
| HLJ225 | | | Fermented sorghum grain | Qiqihaer, Heilongjiang | | CHN FERMENTATION | 12 | 5146 | | | | NF | V121M | A822ins | F | | | x | ✓ | x | ✓ | F | AS I | | PRJNA396809 | Duan et al. 2018 |
| JQ8.3 | | | Fermenting dough | Chengcheng, Shanxi | | CHN FERMENTATION | 12 | 3302 | | | | NF | V121M | | NF | G25del | | x | ✓ | x | ✓ | F | AS I | | PRJNA396809 | Duan et al. 2018 |
| JT49.1 | | | Dry fermented dough | Zhouzhi, Shanxi | | CHN FERMENTATION | 12 | 7371 | | | | NF | V121M | A822ins | NF | G25del | | x | ✓ | x | x | | | | PRJNA396809 | Duan et al. 2018 |
| LJQ13.C7 | | | Fermenting dough | Yanan, Shanxi | | CHN FERMENTATION | 12 | 3044 | | | | NF | V121M | | NF | G25del | | x | ✓ | x | x | | | | PRJNA396809 | Duan et al. 2018 |
| CBS 1544 | PYCC 4654 | *Saccharomyces fructuum* | Fermenting fruit juice | Netherlands | | | | 5165 | | 5/5 | 19/19 | NF | A881del | | NF | 11bp del | | ✓ | x | x | x | | | | PRJEB36095 | This study |
| MTF 2546 | | | Cocoa bean fermentation | West Africa | | COCOA | 13 | 14517 | | 5/5 | | NF | A881del | | F | | | x | x | x | x | | | | ERP014555 | Peter et al. 2018 |
| MTF 2548 | | | Cocoa bean fermentation | West Africa | | COCOA | 13 | 23290 | | 5/5 | | NF | A881del | | F | | | x | x | x | x | | | | ERP014555 | Peter et al. 2018 |
| MTF 2551 | | | Cocoa bean fermentation | West Africa | | COCOA | 13 | 21583 | | 5/5 | | NF | A881del | | F | | | x | x | x | ✓ | NF | AF II | | ERP014555 | Peter et al. 2018 |
| BJ10 | | | Bark of Ulmus sp., secondary forest | Wuling Mountain, Beijing | TEMP | CHN VIII | 14 | 2086 | | 5/5 | | NF | A881del | | F | | | x | x | x | x | | | | PRJNA396809 | Duan et al. 2018 |
| BJ2 | | | Apple, orchard | Shunyi, Beijing | TEMP | CHN VIII | 14 | 2647 | | 5/5 | | F | | | F | | | x | x | x | x | | | | PRJNA396809 | Duan et al. 2018 |
| CBS 1598 | | | Sake moto | Nakazawa, Japan | | SAKE | 15 | 3517 | | | | NF | V121M | | NF | G25del | | ✓ | ✓ | x | x | | | | ERS1108639 | Gonçalves et al. 2016 |
| CBS 435 | PYCC 8194 | *Saccharomyces tokyo* | Sake moto | Nakazawa, Japan | | SAKE | 15 | 4906 | | | | NF | V121M | | NF | G25del | | ✓ | ✓ | x | x | | | | ERS1108637 | Gonçalves et al. 2016 |
| UC 5 | UCD612 | | Sene sake | Kurashi, Japan | | SAKE | 15 | n.d. | | | | NF | V121M | | NF | G25del | | x | ✓ | x | x | | | | PRJNA60197 | Justin Fay, Washington University |
| Kyokai-no.7 | K7 | | Japanese sake brewerie | Japan | | SAKE | 15 | n.d. | | | | NF | V121M | A822ins | NF | G25del | | x | TRIP | x | x | | | | PRJNA45827 | Akao et al. 2011 |
| CBS 1508 | PYCC 5182 | *Saccharomyces carlsbergensis var. mandschuricus* | Starter for sorghum brandy | Unknown | | SAKE | 15 | 1898 | | | | NF | V121M | A822ins | NF | G25del | | ✓ | ✓ | x | ✓ | F | AS I | | ERP014555 | Peter et al. 2018 |
| CBS 440 | | *Saccharomyces formosensis* | Molasses | Taiwan | | SAKE | 15 | 2410 | | 5/5 | | NF | A881del | | NF | G25del | | x | ✓ | x | x | | | | ERP014555 | Peter et al. 2018 |
| Y12 | NRRL Y-12663, CBS 400 | | Fermentation (palm wine) | Ivory Coast | | SAKE | 15 | 15534 | | | | NF | V121M | | NF | G25del | | ✓ | ✓ | x | x | | | | SGRP2 | Bergström, A. et al. 2014 |
| CLIB 1414 | | | Rice wine | Laos | | SAKE | 15 | 11848 | | | | NF | V121M | A822ins | NF | G25del | | ✓ | x | x | x | | | | ERP014555 | Peter et al. 2018 |
| MTJZ1 | | | Fermented sorghum grain | Renhuai, Guizhou, China | | SAKE | 15 | 2515 | | | | NF | V121M | | NF | G25del | | x | ✓ | x | ✓ | F | AS I | | PRJNA396809 | Duan et al. 2018 |
| YJSH1 | | | Bioethanol-producing strain | China | | SAKE | 15 | 3084 | | | | NF | V121M | | NF | G25del | | x | ✓ | x | ✓ | F | AS I | | PRJNA72403 | Zheng. et al. 2012 |
| NRRL Y-12844 | | | Budod (rice-based starter) | Phillipines | | SAKE | 15 | 10363 | | | | NF | V121M | | NF | G25del | | x | ✓ | x | x | | | | ERS1108645 | Gonçalves et al. 2016 |
| NRRL Y-7327 | PYCC 8032 | | Tibetan beer starter | H. Douglas, University of Washington, Seattle, Washington | | SAKE | 15 | 31305 | | | | NF | V121M | C625ins | NF | G25del | | ✓ | ✓ | x | x | | | | PRJEB36095 | This study |
| NRRL Y-6297 | PYCC 8030 | | Coconut tuba (palm wine) | Hipolito, NIST, Manila, Phillipines | TROP | PHILIPPINES | 16 | 3044 | | | | NF | V121M | | NF | G25del | | ✓ | ✓ | x | x | | | | PRJEB36095 | This study |
| YJM 1479 | NRRL Y-6297 | | Coconut tuba (palm wine) | Philippines | TROP | PHILIPPINES | 16 | 3264 | | | | NF | V121M | | NF | G25del | | ✓ | ✓ | x | x | | | | PRJNA189929 | Strope et al. 2015 |
| NRRL YB-4083 | | | Coconut sap | Philippines | TROP | PHILIPPINES | 16 | n.d. | | | | NF | V121M | | NF | G25del | | ✓ | ✓ | x | x | | | | PRJEB36095 | This study |
| YJM 1400 | NRRL YB-4081 | | Guava (fruit) | Philippines | TROP | PHILIPPINES | 16 | 2960 | | | | NF | V121M | | NF | G25del | | x | ✓ | x | x | | | | PRJNA189911 | Strope et al. 2015 |
| CBS 1576 | | | Exudate of *Arenga* sp. (palm tree) | Celebes, Sulavesi, Indonesia | TROP | PHILIPPINES | 16 | 2576 | | | | NF | V121M | | F | | | ✓ | x | x | x | | | | ERP014555 | Peter et al. 2018 |
| NRRL Y-5508 | PYCC 8029 | | Coconut pod | Arguelles, Manila, Philippines | TROP | PHILIPPINES | 16 | 19036 | | | | NF | V121M | | F | | | ✓ | ✓ | x | x | | | | PRJEB36095 | This study |
| DJ66 | | | Palm wine | Yoboki, Djibouti | TROP | PHILIPPINES | 16 | 19012 | | | | NF | T178 del | A822ins | F | | | DUP | ✓ | x | ✓ | NF | AF II | | ERP014555 | Peter et al. 2018 |
| S8BM-30-2D | | | Phloem sap of *Caryota urens* (Toddy palm) | Sri Lanka | TROP | PHILIPPINES | 16 | 3172 | | | | NF | V121M | | F | | | ✓ | ✓ | x | ✓ | F | AA | | ERP014555 | Peter et al. 2018 |
| CBS 2992 | | | Palm wine | Pakistan | TROP | PHILIPPINES | 16 | 2405 | | | | NF | V121M | A822ins | NF | G25del | | ✓ | x | x | x | | | | PRJEB36095 | This study |
| CBS 3000 | | | Palm wine | Pakistan | TROP | PHILIPPINES | 16 | 2325 | | | | NF | V121M | | NF | G25del | | ✓ | ✓ | x | x | | | | PRJEB36095 | This study |
| SDO 3s1 | | | Oak | North Carolina, USA | TEMP | NA & JP - CHN VI/VII - FER | 17 | 3693 | | | | F | | | F | | | x | x | x | x | | | | PRJEB7601 | Almeida et al. 2015 |
| YPS 1009 | | | Oak exudate | New Jersey, USA | TEMP | NA & JP - CHN VI/VII - FER | 17 | 2650 | | | | F | | | F | | | x | x | x | x | | | | PRJNA60223 | Justin Fay, Washington University |
| YPS 163 | | | *Quercus rubra* | Pennsylvania, USA | TEMP | NA & JP - CHN VI/VII - FER | 17 | 2673 | | | | F | | | F | | | x | x | x | x | | | | PRJNA28813 | Doniger et al. 2008 |
| ZP 651 | | | *Quercus acutissima* | Chiba Prefecture, Japan | TEMP | NA & JP - CHN VI/VII - FER | 17 | 3623 | | | | F | | | F | | | x | x | x | x | | | | PRJEB7601 | Almeida et al. 2015 |
| IY_03-5-30-1-1-1_(1) | | | *Chauliodes pectinicornis* (male) - Corydalidae | Louisiana, USA | TEMP | NA & JP - CHN VI/VII - FER | 17 | 2782 | | | | F | | | F | | | x | x | x | x | | | | ERP014555 | Peter et al. 2018 |
| ZP 652 | | | *Quercus acutissima* | Chiba Prefecture, Japan | TEMP | NA & JP - CHN VI/VII - FER | 17 | 3699 | | | | F | | | F | | | x | x | x | x | | | | PRJEB7601 | Almeida et al. 2015 |
| ZP 779 | | | *Quercus acutissima* | Hirusen highland, Okayama Prefecture, Japan | TEMP | NA & JP - CHN VI/VII - FER | 17 | 3509 | | | | F | | | F | | | x | x | x | x | | | | PRJEB7601 | Almeida et al. 2015 |
| ZP 781 | | | *Quercus serrata* | Hirusen highland, Okayama Prefecture, Japan | TEMP | NA & JP - CHN VI/VII - FER | 17 | 3333 | | | | F | | | F | | | x | x | x | x | | | | PRJEB7601 | Almeida et al. 2015 |
| BJ28 | | | Bark of *Castanea* sp., secondary forest | Wuling Mountain, Beijing | TEMP | NA & JP - CHN VI/VII - FER | 17 | 8648 | | 3/5 | | F | | | F | | | x | x | x | x | | | | PRJNA396809 | Duan et al. 2018 |
| FJ12 | | | Peach from a market | Wuyi Mountain, Fujian | TEMP | NA & JP - CHN VI/VII - FER | 17 | 3074 | | | | F | | | F | | | x | x | x | x | | | | PRJNA396809 | Duan et al. 2018 |
| HLJ2 | | | Bark of *Quercus mongolica* , secondary forest | Jingbo lake, Heilongjiang | TEMP | NA & JP - CHN VI/VII - FER | 17 | 2803 | | | | F | | | F | | | x | x | x | x | | | | PRJNA396809 | Duan et al. 2018 |
| N163.01-5A | | | Exudate of *Quercus mongolica* | Botanical garden, Blagoveshchensk, Russia | TEMP | NA & JP - CHN VI/VII - FER | 17 | 3049 | | | | F | | | F | | | x | x | x | x | | | | ERP014555 | Peter et al. 2018 |
| N3.00-7A | | | Grape berries of wild *Vitus amurensis* | Botanical garden, Blagoveshchensk, Russia | TEMP | NA & JP - CHN VI/VII - FER | 17 | 2432 | | | | F | | | F | | | x | x | x | x | | | | ERP014555 | Peter et al. 2018 |
| N159.01-2B | | | Exudate of *Quercus mongolica* | Botanical garden, Blagoveshchensk, Russia | TEMP | NA & JP - CHN VI/VII - FER | 17 | 2863 | | | | F | | | F | | | x | x | x | x | | | | ERP014555 | Peter et al. 2018 |
| SD1 | | | Persimmon, planted tree | Anqiu, Shandong | TEMP | CHN VI / CHN VII | 18 | 2095 | | | | F | | | F | | | x | x | x | x | | | | PRJNA396809 | Duan et al. 2018 |
| SX10 | | | Persimmon, planted tree | Qinling Mountain, Shaanxi | TEMP | CHN VI / CHN VII | 18 | 2509 | | | | F | | | F | | | x | x | x | x | | | | PRJNA396809 | Duan et al. 2018 |
| XJ1 | | | Soil, orchard | Xinjiang | TEMP | CHN VI / CHN VII | 18 | 2880 | | | | F | | | F | | | x | x | x | x | | | | PRJNA396809 | Duan et al. 2018 |
| CBS 403 | PYCC 4571 | *Saccharomyces lindneri* | Ginger beer from *Zingiber officinale* | West Africa | | WEST AFRICA | 19 | 2031 | | | | NF | A881del | | F | | | ✓ | ✓ | x | ✓ | NF | AF II | | ERP014555 | Peter et al. 2018 |
| YJM 1439 | NCYC 110 | | Ginger beer from *Zingiber officinale* | West Africa | | WEST AFRICA | 19 | 2129 | | | | F | | | F | | | ✓ | ✓ | x | ✓ | NF | AF II | | PRJNA189920 | Strope et al. 2015 |
| PYCC8489 | NRRL Y-12633; CBS 400 | *Saccharomyces chevalieri* | Palm wine from *Elaeis guineensis* | Ivory Coast | | WEST AFRICA | 19 | 3572 | | | | NF | A881del | | F | | | ✓ | ✓ | x | ✓ | NF | AF II | | PRJEB36095 | This study |
| YJM 1248 | NRRL Y-1546 | | Bili wine from *Osbeckia grandiflora* | West Africa | | WEST AFRICA | 19 | 2600 | | | | NF | A881del | | F | | | ✓ | ✓ | x | ✓ | NF | AF II | | PRJNA189888 | Strope et al. 2015 |
| PW5 | | | Raphia palm wine | Aba, Abia state, Nigeria | | WEST AFRICA | 19 | 1175 | | | | NF | A881del | | F | | | ✓ | x | x | ✓ | NF | AF II | | PRJNA60181 | Justin Fay, Washington University |
| YJM 195 | NCYC 762 | | Palm wine | Nigeria | | WEST AFRICA | 19 | 2977 | | | | NF | A881del | | F | | | ✓ | ✓ | x | x | | | | PRJNA189849 | Strope et al. 2015 |
| CBS 7903 | NCYC 761 | | Palm wine | Nigeria | | WEST AFRICA | 19 | 2737 | | | | NF | A881del | | F | | | ✓ | ✓ | x | x | | | | PRJEB36095 | This study |
| MUCL_30909-2C | | | Cassava | Burundi | | WEST AFRICA | 19 | 3847 | | 5/5 | | F | | | NF | 11bp del | | ✓ | x | x | x | | | | ERP014555 | Peter et al. 2018 |
| YN6 | | | Bark of *Castanopsis orthacantha* , suburban tree | Zixi Mountain, Yunnan | | | | 2463 | | | | F | | | F | | | x | x | x | x | | | | PRJNA396809 | Duan et al. 2018 |
| CLQCA 20-156 | | | Flower (*Gesneriaceae* sp.) | Yasuni, Orellana, Ecuador | TROP | WB1 - CHN V | 20 | 2991 | | | | F | | | F | | | x | x | x | ✓ | F | AS I | | ERP014555 | Peter et al. 2018 |
| CLQCA 20-314 | | | Termite mound | Yasuni, Orellana, Ecuador | TROP | WB1 - CHN V | 20 | 3468 | | | | F | | | F | | | x | x | x | ✓ | F | AS I | | ERP014555 | Peter et al. 2018 |
| UFMG-CM-Y455 | C45 | | *Tapirira guianensis* | Tocantins, Brazil | TROP | WB1 - CHN V | 20 | 4167 | | | | F | | | F | | | x | x | x | ✓ | F | AS I | SA | PRJEB11698 | Barbosa et al. 2016 |
| UFMG-CM-Y461 | C145 | | Mushroom | Roraima, Brazil | TROP | WB1 - CHN V | 20 | 3675 | | | | F | | | F | | | x | x | x | ✓ | F | AS I | SA | PRJEB11698 | Barbosa et al. 2016 |
| UFMG-CM-Y650 | TOC1364 | | *Tapirira guianensis* | Tocantins, Brazil | TROP | WB1 - CHN V | 20 | 2373 | | | | F | | | F | | | x | ✓ | x | ✓ | F | AS I | SA | PRJEB11698 | Barbosa et al. 2016 |
| ZJ1 | | | Rotten wood, primeval forest | Gutian Mountain, Zhejiang province, China | TROP | WB1 - CHN V | 20 | 929 | | | | F | | | F | | | x | ✓ | x | ✓ | F | AS I | | PRJNA396809 | Duan et al. 2018 |
| HN15 | | | Rotten wood | Wuzhi Mountain, Hainan province, China | TROP | WB1 - CHN V | 20 | 3077 | | | | F | | | F | | | x | ✓ | x | ✓ | F | AS I | | ERP014555 | Peter et al. 2018 |
| HN16 | | | Soil | Wuzhi Mountain, Hainan province, China | TROP | WB1 - CHN V | 20 | 2933 | | | | F | | | F | | | x | ✓ | x | ✓ | F | AS I | | ERP014555 | Peter et al. 2018 |
| GT 39.1 | | | Bark, primeval forest | Gutian Mountain, Zhejiang province, China | TEMP | CHN X | 21 | 969 | | | | F | | | F | | | x | ✓ | x | ✓ | F | AS II | | PRJNA396809 | Duan et al. 2018 |
| JXXY1.1 | | | Bark, primeval forest | Xiangxiyuan, Shennongjia, Hubei province, China | TEMP | CHN X | 21 | 955 | | | | F | | | F | | | x | ✓ | x | ✓ | F | AS II | | PRJNA396809 | Duan et al. 2018 |
| ZP 1273 | | | Soil | New Caledonia | TROP | CHN X | 21 | 3776 | | | | F | | | F | | | ✓ | x | x | x | | | | PRJEB36095 | This study |
| YJM 1447 | UWOPS 05-227.2 | | Bertram palm | Malaysia | TROP | MALAYSIA | 22 | n.d. | | | | NF | 106bp del | | NF | G528del | | ✓ | ✓ | x | ✓ | F | AS I | | PRJNA189923 | Strope et al. 2015 |
| UWOPS 03-433.3 | | | Nectar of Bertram palm | Malaysia | TROP | MALAYSIA | 22 | 1908 | | | | NF | 106bp del | | NF | G528del | | ✓ | ✓ | x | ✓ | F | AS I | | ERS1108647 | Gonçalves et al. 2016 |
| UWOPS 03-461.4 | | | Nectar of Bertram palm | Malaysia | TROP | MALAYSIA | 22 | 18636 | | | | NF | 106bp del | | NF | G528del | | ✓ | ✓ | x | ✓ | F | AS I | | SGRP2 | Bergström et al. 2014 |
| HN10 | | | Rotten wood | Wuzhi Mountain, Hainan province, China | TROP | CHN III | 23 | 4751 | | | | F | | | F | | | x | ✓ | x | ✓ | F | AS I | | ERP014555 | Peter et al. 2018 |
| HN19 | | | Bark from tree in Fagaceae family | Wuzhi Mountain, Hainan province, China | TROP | CHN III | 23 | 2896 | | | | F | | | F | | | x | ✓ | x | ✓ | F | AS I | | ERP014555 | Peter et al. 2018 |
| HLJ1 | | | Bark of *Quercus mongoLica* , secondary forest | Jingbo lake, Heilongjiang | TEMP | CHN IV | 24 | 2865 | | | | F | | | F | | | x | x | x | x | | | | PRJNA396809 | Duan et al. 2018 |
| N51-1A | | | Exudate of *Quercus mongolica* | Vladivostok, Russian Far East | TEMP | CHN IV | 24 | 3074 | | | | F | | | F | | | x | x | x | x | | | | ERP014555 | Peter et al. 2018 |
| BJ21 | | | Bark of *Carya cathayensis* , secondary forest | Dongling Mountain, Beijing | TEMP | CHN IV | 24 | 3286 | | | | F | | | F | | | x | x | x | x | | | | PRJNA396809 | Duan et al. 2018 |

| | | | | | | | | | | | | | | | | | | | | | | | | | | |
|---|---|---|---|---|---|---|---|---|---|---|---|---|---|---|---|---|---|---|---|---|---|---|---|---|---|---|
| BJ14 | | | Forest soil, secondary forest | Wuling Mountain, Beijing | TEMP | CHN IV | 24 | 2922 | | | | F | | | F | | | x | x | x | x | | | | PRJNA396809 | Duan et al. 2018 |
| BJ6 | | | Persimmon | Changping, Beijing, China | TEMP | CHN IV | 24 | 3068 | | | | F | | | F | | | x | x | x | x | | | | ERP014555 | Peter et al. 2018 |
| HN2 | | | Rotten wood, primeval forest | Diaoluo Mountain, Hainan province, China | TROP | CHN I | 25 | 3283 | | | | F | | | F | | | x | ✓ | x | ✓ | F | AS I | | PRJNA396809 | Duan et al. 2018 |
| HN6 | | | Rotten wood, primeval forest | Wuzhi Mountain, Hainan province, China | TROP | CHN I | 25 | 2780 | | | | F | | | F | | | x | ✓ | x | x | | | | ERP014555 | Peter et al. 2018 |
| JXXY16.1 | | | Bark, primeval forest | Xiangxiyuan, Shennongjia, Hubei province, China | TEMP | CHN IX | 26 | 1234 | | | | F | | | F | | | x | ✓ | x | x | | | | PRJNA396809 | Duan et al. 2018 |
| XXY30L.2 | | | Bark, primeval forest | Xiangxiyuan, Shennongjia, Hubei province, China | TEMP | CHN IX | 26 | 3191 | | | | F | | | F | | | x | ✓ | x | x | | | | PRJNA396809 | Duan et al. 2018 |
| XXYS1.4 | | | Bark, primeval forest | Xiangxiyuan, Shennongjia, Hubei province, China | TEMP | CHN IX | 26 | 992 | | | | F | | | F | | | x | ✓ | x | x | | | | PRJNA396809 | Duan et al. 2018 |
| SX3 | | | Bark from *Carya* sp. | Qinling Mountain, Shaanxi province, China | TEMP | CHN II | 27 | 2833 | | | | F | | | F | | | x | x | x | x | | | | ERP014555 | Peter et al. 2018 |
| SX1 | | | Persimmon, planted tree | Qinling Mountain, Shaanxi province, China | TEMP | CHN II | 27 | 2509 | | | | F | | | F | | | x | x | x | x | | | | ERP014555 | Peter et al. 2018 |
| SX8 | | | Bark of *Quercus fabri* , primeval forest | Qinling Mountain, Shaanxi province, China | TEMP | CHN II | 27 | 2602 | | | | F | | | F | | | x | x | x | x | | | | PRJNA396809 | Duan et al. 2018 |

[1] Count of heterozygous sites across the genome. Values higher than 20.000 are highlighted.
[2] Three genomic regions horizontally transferred from non-*Saccharomyces* yeasts (Novo et al. 2009) that encompass genes potentially relevant for the winemaking process.
[3] Aquaporins (encoded by *AQY1* and *AQY2* ) are membrane water channels that facilitate the transport of water in and out of the cell. Their inactivation is viewed as a consequence of domestication.
[4] Subtelomeric gene associated with the locus of sucrose utilization that provides resistance to inhibitory compounds present in molasses.
[5] These genes encode enzymes involved in the synthesis of biotin and were originally found in strains used in sake fermentation.
[6] Chimeric gene that gives rise to an extra-cellular glucoamylase that allows the conversion of soluble starch and dextrins into fermentable sugars.
[7] Polymorphic genes that encode an α-galactosidase that hydrolyses melibiose.

TROP -Tropical
TEMP -Temperate

CHN - China
FER - Far East Russia
FG - French Guiana
MA - Mexican agavae fermentation
MO - Mediterranean oak
NA & JP - North America and Japan
PDM - Prise de mousse
WB - Wild Brazil

F Functional
NF Not Functional

x Absent
✓ Present

✓ ✓ *S. cerevisiae*
✓ *S. paradoxus*
✓ Unknown

AS I - ASIAN I
AS II - ASIAN II
AA - ASIAN - AMERICAN
R - RECOMBINANT ASII-AA
EUR - EUROPEAN
AF I - AFRICAN I
AF II - AFRICAN II
SA - SOUTH AMERICAN

AQY1 INACTIVATIONS

| | |
|---|---|
| A881del | adenine deletion at position 881 |
| V121M | valine to methionine at aminoacid 121 |
| A822ins | adenine insertion at position 822 |
| 321 G>A Stop | guanine to adenine at position 321 leads to a premature stop codon |
| T498del | thymine deletion at position 498 |
| T239 del | thymine deletion at position 239 |
| T230 del | thymine deletion at position 230 |
| 88 C>T Stop | cytosine to thymine at position 88 leads to a premature stop codon |
| A863del | adenine deletion at position 863 |
| C625ins | cytosine insertion at position 625 |
| T178 del | thymine deletion at postion 178 |
| 106bp del | deletion of the first 106 bp |

AQY2 INACTIVATIONS

| | |
|---|---|
| 11bp del | 11 bp deletion |
| G25del | guanine deletion at position 25 |
| 20bp ins | 20 bp insertion |
| 786 G>A stop | guanine to adenine at position 786 leads to a premature stop codon |
| 424 C>T Stop | cytosine to thymine at position 424 leads to a premature stop codon |
| GT158ins | guanine and thymine insertion at position 158 |
| G107 del | guanine deletion at position 107 |
| 185 T>A Stop | thymine to adenine at position 185 leads to a premature stop codon |
| 792stop | premature stop codon at position 792 |
| G528del | guanine deletion at position 528 |

References:

Akao et al. 2011. Whole-genome sequencing of sake yeast *Saccharomyces cerevisiae* Kyokai no. 7. DNA Res. 18, 423-434.
Almeida et al. 2015. A population genomic insigth into the Mediterranean origins of wine yeast domestication. Mol. Eco. 24, 5412-5427.
Almeida et al. 2017. Adaptive divergence in wine yeasts and their wild relatives suggests a prominent role for introgressions and rapid evolution at noncoding sites. Mol. Ecol. 26, 2167-2182.
Barbosa et al. 2016. Evidence of natural hybridization in Brazilian wild lineages of *Saccharomyces cerevisiae* . GBE. 8:317-329.
Barbosa et al. 2018. Multiple rounds of artificial selection promote microbe secondary domestication – the case of cachaça yeasts. GBE 10,1939-1955.
Bergström et al. 2014. A high-definition view of functional genetic variation from natural yeast genomes. Mol Biol Evol 31, 872–888.
Borneman et al. 2008. Comparative genome analysis of a *Saccharomyces cerevisiae* wine strain. FEMS Yeast Res. 8, 1185-1195.
Borneman et al. 2011. Whole-genome comparison reveals novel genetic elements that characterize the genome of industrial strains of *Saccharomyces cerevisiae* . PLoS Genet. 7, E1001287.
Coutouné et al. 2017. Draft genome sequence of *Saccharomyces cerevisiae* Barra Grande (BG-1), a Brazilian industrial bioethanol-producing strain. Genome Announc. 5:e00111-17.
Doniger et al. 2008. A catalog of neutral and deleterious polymorphism in yeast. PLoS Genet 4, e1000183.
Duan et al. 2018. The origin and adaptive evolution of domesticated populations of yeast from Far East Asia. Nature communications. 9.1: 2690.
Dunn et al. 2012. Analysis of the *Saccharomyces cerevisiae* pan-genome reveals a pool of copy-number variants distributed in diverse yeast strains from differing industrial environments. Genome Res. 22, 908-924.
Gallone et al. 2016. Domestication and divergenceof *Saccharomyces cerevisiae* beer yeasts. Cell 166, 1397-1410.
Gonçalves et al. 2016. Distinct domestication trajectories in top-fermenting beer yeasts and wine yeasts. Curr Biol 26, 2750-2761.
Legras et al. 2018. Adaptation of *S. cerevisiae* to fermented food environments reveals remarkable genome plasticity and the footprints of domestication. MBE 35(7):1712-1727.
Novo et al. 2009. Eukaryote-to-eukaryote gene transfer events revealed by the genome sequence of the wine yeast *Saccharomyces cerevisiae* EC1118. PNAS 106, 16333-16338.
Peter et al. 2018. Genome evolution across 1,011 *Saccharomyces cerevisiae* isolates. Nature. 556.7701: 339.
Pontes et al. 2019. A quasi-domesticate relic hybrid population of *Saccharomyces cerevisiae* x *S. paradoxus* adapted to olive brine. Frontiers in Genetics. 10: 449.
Strope et al. 2015. The 100-genomes strains, an *S. cerevisiae* resource that illuminates its natural phenotypic and genotypic variation and emergence as an opportunistic pathogen. Genome Res. 25, 762-774.
Zheng. et al. 2012. Genome sequencing and genetic breeding of a bioethanol *Saccharomyces cerevisiae* strain YJS329. BMC Genomics. 13, 479.

**Table S2. Presence (+) or absence (-) of the *MEL* gene in the genomes of *S. paradoxus* from different populations.**

| STRAIN | ORIGIN | POPULATION | MEL |
|---|---|---|---|
| DBN13 | Greece | EUROPEAN | + |
| DBN14 | Greece | EUROPEAN | + |
| DBN15 | Greece | EUROPEAN | + |
| TAX15c | Greece | EUROPEAN | - |
| DBN10 | Greece | EUROPEAN | - |
| TAX3d | Greece | EUROPEAN | - |
| TAX2 | Greece | EUROPEAN | - |
| PAR10c | Greece | EUROPEAN | - |
| TAX1a | Greece | EUROPEAN | - |
| ZP 1157 | Portugal | EUROPEAN | - |
| ZP 1023 | Portugal | EUROPEAN | - |
| ZP600 | Portugal | EUROPEAN | - |
| CET10308 | Spain | EUROPEAN | - |
| ZP 1167 | Spain | EUROPEAN | - |
| ZP 1233 | Spain | EUROPEAN | - |
| MB13a | France | EUROPEAN | - |
| MB11d | France | EUROPEAN | - |
| MB15 | France | EUROPEAN | - |
| MB4 | France | EUROPEAN | - |
| Chr116 | France | EUROPEAN | - |
| DBVPG 4650 | Italy | EUROPEAN | - |
| CRO2d | UK | EUROPEAN | - |
| OAK11c | UK | EUROPEAN | - |
| CRO12d | UK | EUROPEAN | - |
| Q59 | UK | EUROPEAN | - |
| Q89 | UK | EUROPEAN | - |
| DBF01 | UK | EUROPEAN | - |
| CBS 406 | Netherlands | EUROPEAN | - |
| ZP 614 | Germany | EUROPEAN | - |
| ZP 665 | Germany | EUROPEAN | - |
| EM11 | Germany | EUROPEAN | - |
| EM13 | Germany | EUROPEAN | - |
| EM55 | Germany | EUROPEAN | - |
| EM213 | Germany | EUROPEAN | - |
| NBRC 102004 | Finland | EUROPEAN | - |
| ALKO2639 | Finland | EUROPEAN | - |
| NBRC 102003 | Lithuania | EUROPEAN | - |
| CBS 5829 | Denmark | EUROPEAN | - |
| CBS 432 | Unknown | EUROPEAN | - |
| EXF 6618 | Slovenia | EUROPEAN | - |
| EXF 6641 | Slovenia | EUROPEAN | - |
| EXF 6729 | Slovenia | EUROPEAN | - |
| EXF 6635 | Slovenia | EUROPEAN | - |
| INMIV11 | Ukraine | EUROPEAN | - |
| INMIV544 | Ukraine | EUROPEAN | - |
| ZP 703 | USA | NORTH AMERICAN | - |
| YPS 125 | USA | NORTH AMERICAN | - |
| YPS 138 | USA | NORTH AMERICAN | - |
| YPS 644 | USA | NORTH AMERICAN | - |
| UCD-FST 61-248 | USA | NORTH AMERICAN | + |
| UCD 51-137 | USA | NORTH AMERICAN | - |
| UCD-72-129 | USA | NORTH AMERICAN | - |
| UWOPS 1979-129 | Canada | NORTH AMERICAN | - |
| UWOPS 1979-527 | Canada | NORTH AMERICAN | - |
| UFRJ 50816 | Brazil | NORTH AMERICAN | - |
| NRLLY2047 | USA | NORTH AMERICAN | - |
| UWOPS 91-917-1 | Hawaii | HAWAIIAN | + |
| CBS 8440 | Russia | FAR EAST | - |
| CBS 8443 | Russia | FAR EAST | - |
| KPN 3829 | Siberia | FAR EAST | - |
| IFO 1804 | Japan | FAR EAST | - |
| UCD-67-570W | Japan | FAR EAST | - |
| N16 | Russia | FAR EAST | - |
| N43 | Russia | FAR EAST | - |
| N44 | Russia | FAR EAST | - |
| N12 | Azerbeijan | FAR EAST | - |
| | | | |
| ZP 811 | | | - |
| ZP 751 | | | - |
| ZP 838 | | | - |
| DBH58 | | | - |

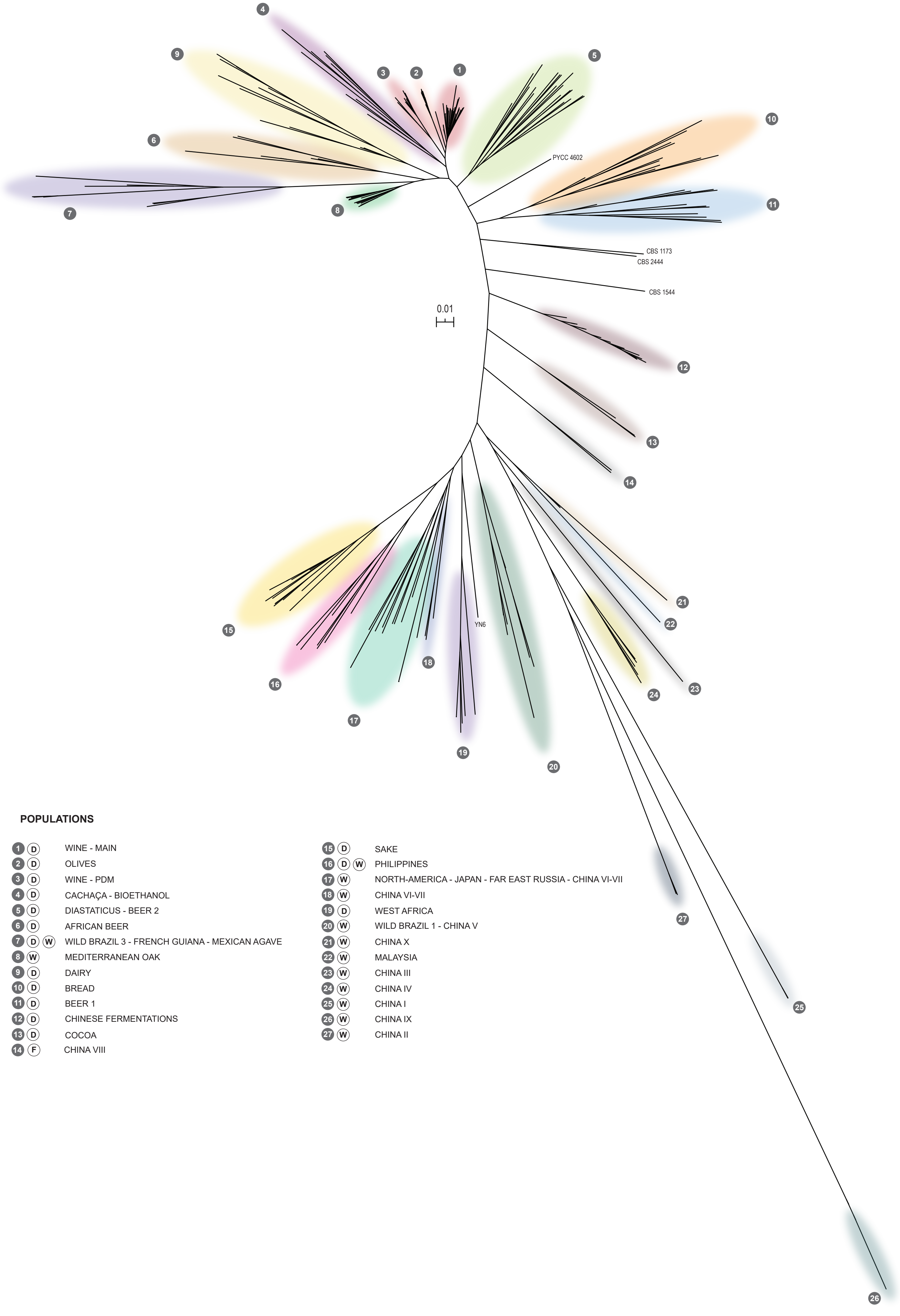


**Figure S1.** Unrooted phylogeny *S. cerevisiae* genomes representing the known diversity of the species. The phylogeny was inferred from 247 sequences and 983.701 SNPS using the TVM+F+G4 model of sequence evolution and the maximum likelihood method as implemented in IQ-TREE. Branch lengths correspond to the expected number of substitutions per site. The 27 clades detected are numbered in gray circles.

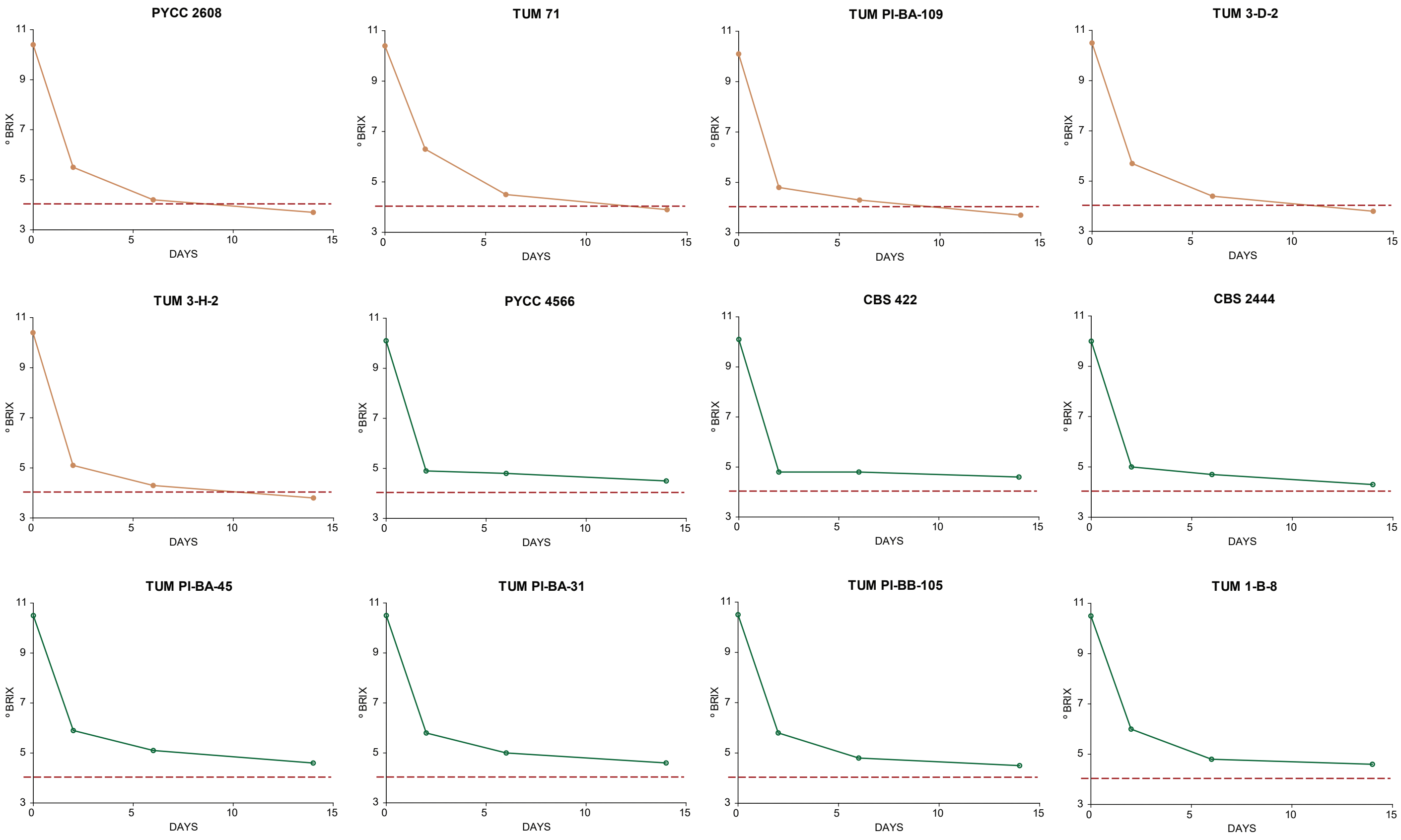


**Figure S2.** °Brix measurements during growth in beer wort for 15 days at 25 °C. Strains able to attenuate the initial 11°Brix of the medium to 4°Brix or lower were considered as diastase positive.

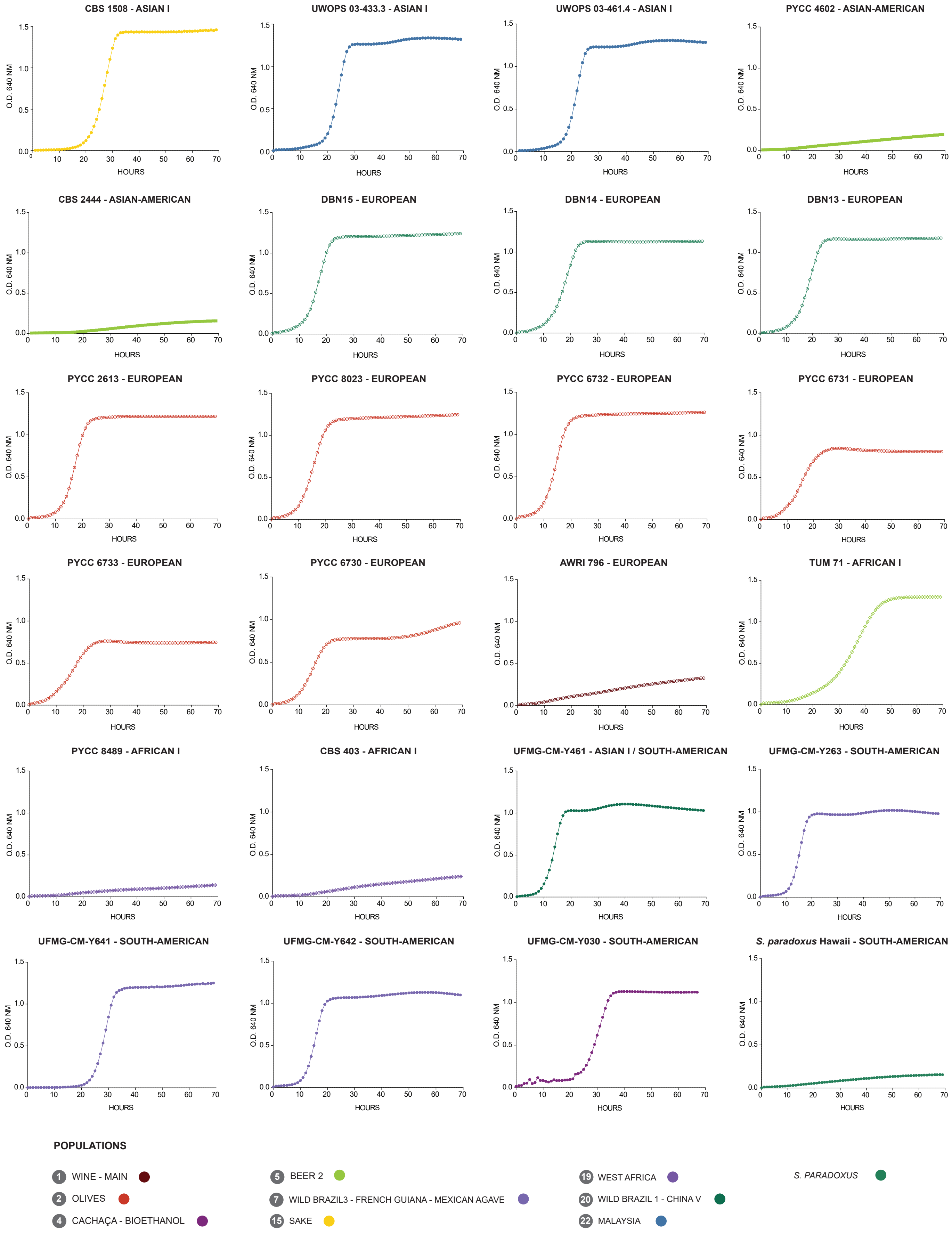


**Figure S3.** Growth curves on 1% (w/v) melibiose medium supplemented with yeast nitrogen base and incubated at 25 °C for three days.
